# _The Homeobox gene, *HOXB13*, Regulates a Mitotic Protein-Kinase Interaction Network in Metastatic Prostate Cancers_

**DOI:** 10.1038/s41598-019-46064-4

**Published:** 2019-07-04

**Authors:** Jiqiang Yao, Yunyun Chen, Duy T. Nguyen, Zachary J. Thompson, Alexey M. Eroshkin, Niveditha Nerlakanti, Ami K. Patel, Neha Agarwal, Jamie K. Teer, Jasreman Dhillon, Domenico Coppola, Jingsong Zhang, Ranjan Perera, Youngchul Kim, Kiran Mahajan

**Affiliations:** 1 Department of Biostatistics and Bioinformatics, H. Lee Moffitt Cancer Center and Research Institute, 12902 Magnolia Drive, Tampa, FL USA; 2 Department of Surgery, Washington University in St. Louis, MO, USA; 3 Bioinformatics Core, Sanford Burnham Prebys Medical Discovery Institute, La Jolla, CA USA; 4 Tumor Biology Department, H. Lee Moffitt Cancer Center and Research Institute, Tampa, FL USA; 5 Department of Anatomic Pathology, H. Lee Moffitt Cancer Center and Research Institute, 12902 Magnolia Drive, Tampa, FL USA; 6 Department of Genitourinary Oncology, H. Lee Moffitt Cancer Center and Research Institute, Tampa, FL USA; 7 Analytical Genomics and Bioinformatics, Sanford Burnham Prebys Discovery Institute, Orlando, FL USA

**Keywords:** Urological cancer, Tumour biomarkers

## Abstract

HOXB13, a homeodomain transcription factor, is linked to recurrence following radical prostatectomy. While HOXB13 regulates Androgen Receptor (AR) functions in a context dependent manner, its critical effectors in prostate cancer (PC) metastasis remain largely unknown. To identify HOXB13 transcriptional targets in metastatic PCs, we performed integrative bioinformatics analysis of differentially expressed genes (DEGs) in the proximity of the human prostate tumor-specific AR binding sites. Unsupervised Principal Component Analysis (PCA) led to a focused core HOXB13 target gene-set referred to as HOTPAM9 (**HO**XB13 **T**argets separating **P**rimary **A**nd **M**etastatic PCs). HOTPAM9 comprised 7 mitotic kinase genes overexpressed in metastatic PCs, *TRPM8*, and the heat shock protein *HSPB8*, whose levels were significantly lower in metastatic PCs compared to the primary disease. The expression of a two-gene set, *CIT* and *HSPB8* with an overall balanced accuracy of 98.8% and a threshold value of 0.2347, was sufficient to classify metastasis. HSPB8 mRNA expression was significantly increased following *HOXB13* depletion in multiple metastatic CRPC models. Increased expression of HSPB8 by the microtubule inhibitor Colchicine or by exogenous means suppressed migration of mCRPC cells. Collectively, our results indicate that HOXB13 promotes metastasis of PCs by coordinated regulation of mitotic kinases and blockade of a putative tumor suppressor gene.

## Introduction

Prostate cancer (PC) is a commonly diagnosed cancer among American men^1^. The prognosis of metastatic castration-resistant prostate cancer (mCRPC) is particularly bleak as the disease progresses after an initial response to androgen deprivation therapies (ADT) with the 5-year survival at ~28%^2–5^. While multiple studies have highlighted the role of tyrosine kinases, expression of the variant AR-V7 or AR-interacting epigenetic modifiers in mCRPC survival, recent studies indicate the importance of tissue-specific and developmentally regulated transcription factors in the disease progression^2,6^. Importantly, shared oncogenic transcription factors and epigenetic regulators can reprogram normal cells of distinct epithelial lineage to converge towards cancer cell lineages with similar molecular features underscoring the importance of these drivers in establishing the tumor landscape and promoting metastasis^7–10^.

The *HOX* family is an evolutionarily conserved group of gene clusters that code for transcription factors characterized by the presence of homeodomains^11^. Among these, *HOXB13* has emerged as a critical mediator of CRPC growth through multiple mechanisms, including its ability to modulate the genomic recruitment of the constitutively active AR splice variant AR-V7^12–16^. Further, a germ line mutation in *HOXB13* (G84E) has been identified which is not only associated with an increased risk of familial and hereditary PC, but male carriers also appear more likely to develop the aggressive form of the disease^17,18^.

During mouse embryonic development, *HOXB13* is expressed in the caudal portion of the tail bud, spinal cord and urogenital sinus^19^. It is essential for the differentiation of the secretory cells in the ventral lobes of the prostate^18^. Subsequently, *HOXB13* expression is not completely switched off but is maintained at low levels in adult prostatic tissues^19^. Even at the earliest stages of mouse embryogenesis, a concurrent expression of AR and HOXB13 is observed; however, HOXB13 expression is not dictated by androgen^18–22^. A context dependent role for HOXB13 has been noted in androgen-dependent versus hormone-refractory prostate cancers^20,22,23^. Mechanistically it has been proposed that HOXB13 can function as a tether, collaborator or negative regulator of the Androgen receptor due to its ability to modulate the expression Androgen-regulated genes^11,24^. In prostate cancer cells, the BET bromodomain protein family member, BRD4, epigenetically regulates HOXB13 expression^16^. Moreover, integrative bioinformatics analysis identified an AR independent BRD4-HOXB13 dependent transcriptome as a proliferative gene network involved in cell cycle progression, nucleotide metabolism, and chromatin assembly^5^. Consistently, genetic depletion of HOXB13 or pharmacological blockade significantly impacts the ability of metastatic CRPC cells to from xenograft tumors in castrated immunocompromised mice^16^. Combined, these results underscore the dependency of mCRPCs on HOXB13 regulatory networks particularly when androgen-dependent AR signaling is impacted and is consistent with its requirement in metastasis^22,23^.

Increased *HOXB13* and *AR* expression is a hallmark of advanced primary and bone metastatic PCs^25,26^. Notably, while ~9700 tumor-specific AR binding sites (T-ARBS) were reported to be enriched for the HOXB13/FOXA1 motifs; which of the putative AR or HOXB13 target genes are critical for tumor growth or metastasis is not well-investigated^24^. In this study, to identify the mechanism by which *HOXB13* promotes metastasis, we performed integrative bioinformatics analysis to uncover genes that are significantly impacted as a result of *HOXB13* depletion in a metastatic PC model cell line and are also differentially expressed in human prostate tumors. This integrative analysis approach revealed a previously unknown network of mitotic kinases and a putative tumor suppressor gene whose expression is significantly modulated between primary and metastatic PCs. Collectively, our results indicate that metastasis of prostate cancers is a highly orchestrated event regulated by the transcriptional of activity of the homeobox gene *HOXB13*.

## Results

### A core HOXB13 target gene set, HOTPAM9, can stratify primary from metastatic PCs

**Figure 1:**
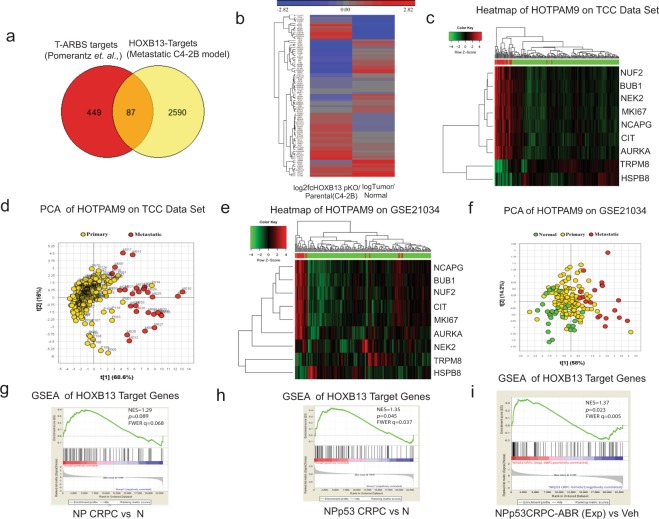
HOXB13 target genes stratify primary from metastatic prostate tumors. **(a)** Venn Diagram of overlaps between DEGS (536 genes) in proximity of the Tumor specific AR binding sites (T-ARBS sites) and DEGS obtained following HOXB13 reduction (2677genes) in HOXB13pKO (n = 2 biological replicates) compared to C4-2B-Parental. **(b)** Heat map comparing expression of the 87 HOXB13 target genes obtained by integrative analysis in (a). Expression values were standardized to mean = 0 and standard deviation = 1 and hierarchically clustered. **(c)** Heat map of microarray analysis showing expression of 9 selected genes (HOTPAM9) in total n = 223 (194 primary and 29 metastatic tumors) in the Moffitt Cancer Center TCC data set. Red bars indicate metastatic tumors. **(d)** Principle Component Analysis (PCA) modeling HOXB13 target genes in 223 PC samples. **(e)** Heat map of prostate tumor microarray analysis showing expression of HOTPAM9 genes in the MSKCC data set (GSE21034). Red bars indicate metastatic tumors. **(f)** PCA analysis modeling HOTPAM9 in 179 samples (29 normal, 131 prostate adenocarcinomas and 19 metastasis). **(g-i)** Gene set enrichment analysis of HOXB13 target genes in castrated *NKX3*.*1*^haplo^(N), *NKX3*.*1*^haplo^ *PTEN*^cnull^ (NP), and *NKX3*.*1*^haplo^ *PTEN*^cnull^ p53^cnull^ (NPp53) genetically engineered mouse models of prostate adenocarcinoma.

HOXB13 is expressed in androgen-dependent LNCaP and VCaP cell lines as well as androgen-independent mCRPC cell lines, C4-2B and 22Rv1^27^. We chose C4-2B for further evaluation as this cell line expresses HOXB13 and AR, shows significant resistance to the anti-androgen Enzalutamide, and metastasizes in castrated immunocompromised male mice^2,16^. To identify HOXB13 transcriptional targets in high-risk metastatic human PCs, we performed integrative bioinformatics analysis of 536 differentially expressed genes (DEGs) in the proximity of the tumor-specific AR binding sites (T-ARBS) enriched for HOXB13/FOXA1 binding motifs^24^ (Supplementary Fig. 1a) with the 2677 DEGs that were found to be significantly affected as a result of HOXB13 reduction in the metastatic CRPC cell line, C4-2B/pHOXB13KO (Fig. 1a)^16^. Integrative bioinformatics analysis yielded 87 HOXB13 specific target genes (HOXTAR87) (Fig. 1a,b) in proximity of the T-ARBS sites. We observed that following *HOXB13* reduction, genes which are highly expressed in C4-2B/pHOXB13KO were downregulated in human prostate tumors while those with reduced expression in C4-2B/pHOXB13KO were upregulated in human prostate tumors compared to normal prostates (Fig. 1b).

To further ascertain the clinical significance of HOXTAR87, we analyzed their expression in the microarray data obtained from 194 localized and 29 metastatic tumors from the Moffitt Cancer Center Total Cancer Care (TCC) dataset (Supporting Data Table 1). Unsupervised Principle Component Analysis (PCA) was performed on Moffitt TCC gene expression data employing the HOXTAR87 genes. We observed separation between the primary and metastatic cancers in the second Principal Component. Subsequently, to identify a minimal gene set driving this separation, we selected genes with the most extreme loadings and identified a 9-gene set that maximized the separation of primary and metastatic cancers when reapplied in the first Principle Component. Heat maps were generated for this 9-gene subset (Fig. 1c; Supporting Data Table 2). The 9 gene-set could effectively distinguish primary organ confined from metastatic PCs by first component of PCA at 56.7% (Fig. 1d). This 9 core HOXB13 target gene set is referred to as the HOTPAM9 (**HO**XB13 **T**arget genes separating **P**rimary and **M**etastatic PCs).

The HOTPAM9 core gene set was then independently validated for its ability to separate primary from metastatic tumors in the publicly available MSKCC data set- 179 samples (29 normal, 131 primaries and 19 metastasis)^28^ (Fig. 1e,f; Supporting Data Table 3). HOTPAM9 gene set was also effective at separating primary tumors from adjacent normal and primary from metastatic cancers (compare Fig. 1d,f; Supporting Data Table 4).

To further ascertain the significance of HOXB13 target genes in additional data sets, we performed meta-analysis of gene expression data from genetically engineered mouse models (*NKX3*.*1*^CreERT2/+^/*PTEN*^flox^ (NP mice) and the NPp53^flox^ (NPp53 mice)^3^. These mice specifically lose prostate-specific expression of *PTEN* and tumor suppressor p53 in the epithelial compartment following tamoxifen treatment and develop CRPCs following castration with molecular features reminiscent of metastatic human CRPCs^3^. Gene Set Enrichment Analysis (GSEA) (Fig. 1g–i) revealed a positive enrichment for the HOXB13 target genes (HOXTAR87) specifically in tumors derived from NPp53 CRPC compared to *NKX3*.*1*^CreERT2/+^ mice (N) (NES = 1.35, *p* = 0.045) (Fig. 1; compare g to h). A significant enrichment of HOXTAR81 was not observed in NP vs N mice (NES = 1.29, *p* = 0.089) (Fig. 1g), NP CRPC vs NP (NES = −0.92, *p* = 0.610), NPp53 CRPC vs Np53 (NES = −0.79, *p* = 0.88), NPp53 CRPC vs NP CRPC (NES = 1.25, *p* = 0.096 (Supplementary Figs 2a–c). In contrast, the NPp53 CRPC mice treated with Abiraterone (ABR), a subset referred as exceptional non-responders due to their propensity to develop accelerated tumor phenotypes and metastasis revealed a significant enrichment (NES = 1.37, *p* = 0.023) (Fig. 1i), suggesting a tendency for these HOXTAR81 genes to regulate a metastatic CRPC program. Together, these results suggest that upregulation of the HOXB13 target gene expression correlates with severity of disease progression and anti-androgen resistance.

### HOTPAM9 genes are transcriptional targets of *HOXB1*3 in PC

**Figure 2:**
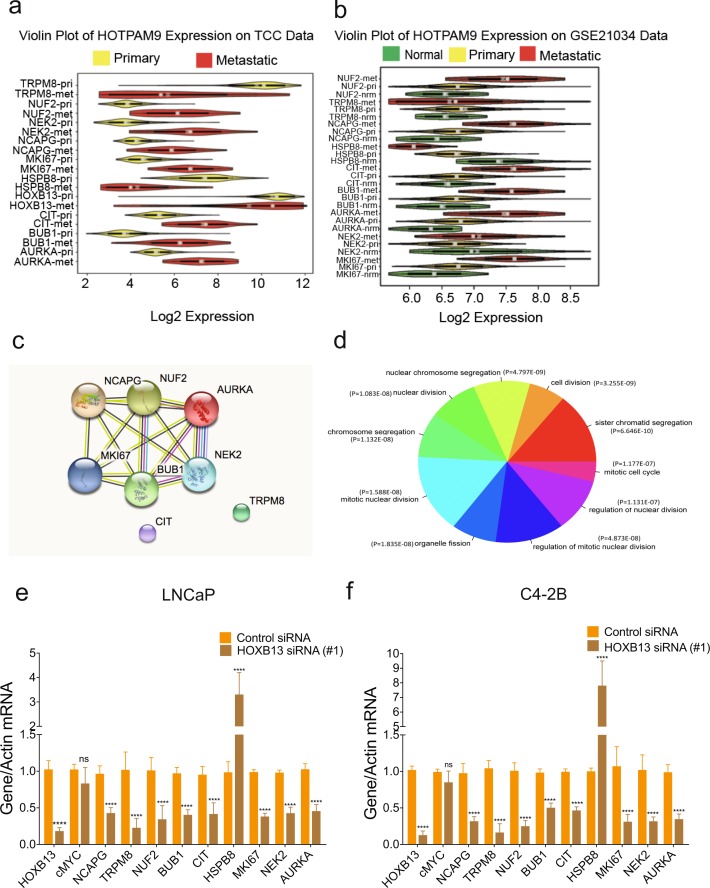
HOTPAM9 comprises a mitotic protein-protein interaction network. **(a)** Violin plots displaying HOTPAM9 gene expression differences in the primary versus metastatic cancers in the Moffitt Cancer Center TCC data set. **(b)** Violin plot of HOTPAM9 genes in MSKCC validation data set. **(c)** STRING analysis of the HOTPAM9 genes indicating Protein-Protein Interaction network (PPI). PPI enrichment *p*-value, 1.47 e-13. **(d)** Gene Ontology (GO) analysis revealed 9 significantly enriched gene sets impacted by HOXB13 depletion. LNCaP **(e)** and C4-2B **(f)** cells were transfected with control or HOXB13 siRNA. Total RNA was isolated, followed by qRT-PCR with HOXB13, c-MYC and HOTPAM9 primers. Data are represented as mean ± SEM and normalized to actin. n = 2 biological replicates, triplicate samples in each biological replicate. ****p < 0.0001, ns: not significant.

Violin plots revealed that a majority (6/9) of the HOTPAM9 metastasis signature were overexpressed in metastatic tumors compared to the primary tumors in the Moffitt TCC data set (Fig. 2a; Supporting Data Table 5) and MSKCC (GSE21034) validation data set (Fig. 2b; Supporting Data Table 6) and GSE6919 (Supplementary Fig. 2d; Supporting Data Tables 7–10). In contrast, expression of the putative tumor suppressor gene, *HSPB8*^29^, was found to be consistently downregulated in multiple clinical data sets (Fig. 2a,b, Supplementary Fig. 2d; Supplementary Data Tables 5, 6 and 10). Search Tool for the Retrieval of Interacting Genes (STRING) analysis revealed that a majority of the HOTPAM9 genes are serine/threonine kinases that cross-talk through protein-protein interactions (PPI enrichment *p-value*: 1.47e-13) to ensure high fidelity chromosome separation during mitosis. These kinases monitor centrosome separation, bipolar spindle assembly, chromosome alignment and cytokinesis (Fig. 2c,d). Pathway analysis revealed a subset of the HOTPAM9 genes (*AURKA*, *BUB1*, *CIT*, *NUF2*, *NEK2*, and *NCAPG*) regulate key phases of cell division (Fig. 2d). Distinct from the HOTPAM9 kinases and not a part of the network is the Transient Receptor Potential cation channel subfamily member M8, TRPM8, which regulates PC cell migration, and hence suggests a role in tumor metastasis (Fig. 2c; Supplementary Fig. 1b)^30,31^.

To confirm that the HOTPAM9 genes are regulated in a HOXB13-dependent manner PC cell lines^27^ C4-2B, LNCaP, VCaP and PC3 were transfected with control or HOXB13 silencing RNAs either singly or pooled (siRNAs). After 48 hours, the relative expression of HOTPAM9, HOXB13 and c-MYC was examined by quantitative Reverse Transcriptase PCR (qRT-PCR) with gene-specific primers. We observed that expression of a majority of the HOTPAM9 kinase expression was significantly impacted when *HOXB13* expression was silenced with HOXB13 siRNAs in C4-2B and LNCaP and in VCaP cells to a certain extent (BUB1, NEK2, and AURKA) growing in charcoal stripped media, but not in the small cell carcinoma cell line PC3 (Fig. 2e,f, Supplementary Fig. 2e). In contrast, the expression of *HSPB8* gene was significantly upregulated in both AR positive cell lines (LNCaP, C4-2B, and VCaP) as well as an AR negative cell line (PC3) (Fig. 2e,f, Supplementary Fig. 2e). c-MYC was used as a negative control and its expression remains unchanged following HOXB13 silencing in AR positive cell lines but was upregulated in the PC3 (Fig. 2e,f; Supplementary Fig. 3b). Silencing of HOXB13 also impacted AR/AR-V7, PSA and NKX3.1 in VCaP and C4-2B but not TMPRSS2 in VCaP (Supplementary Fig. 3a,b). NKX3.1 is a previously characterized HOXB13/AR target gene and was down regulated in both models (Supplementary Fig. 3a,b)^11^.

**Figure 3:**
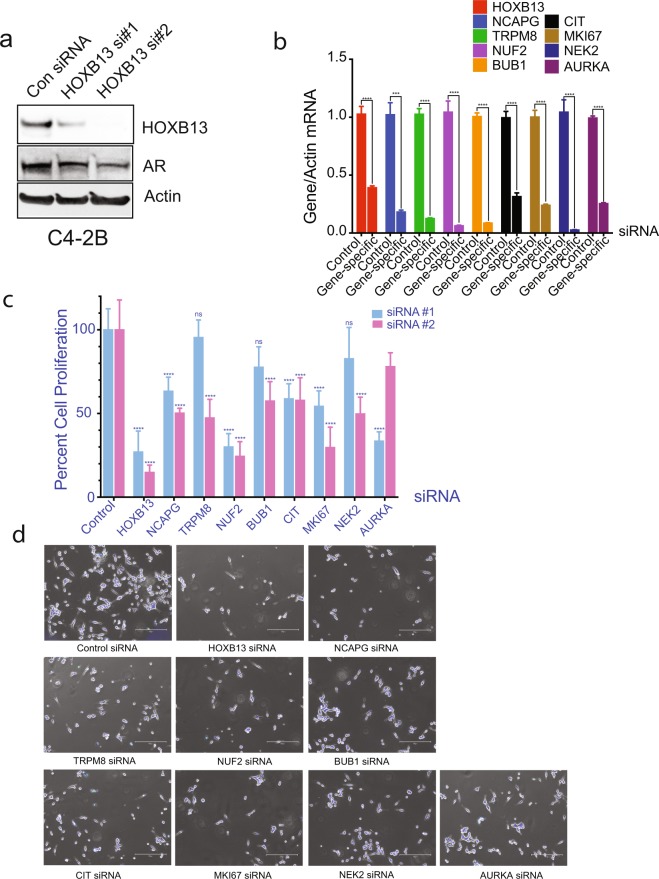
Effect of HOXB13 and HOTPAM9 gene silencing on mCRPC proliferation. **(a)** C4-2B cells were transfected with control or HOXB13 siRNAs (#1 is individual and #2 is pooled). Whole cell lysates were immunoblotted with anti-HOXB13 or anti-AR antibodies. Actin is a normalization control. **(b**–**d)** C4-2B cells were transfected with control or HOXB13 or HOTPAM9 siRNAs. **(b)** Total RNA was isolated, followed by qRT-PCR with primers corresponding to the transfecting siRNA. Actin is a normalization control. **(c)** Cells were harvested, stained with Trypan blue, and counted using a hemocytometer 96 h post-transfection. siRNAs #1 data represent n = 2 biological replicates, duplicate samples in each biological replicate. **(d)** Cells were stained with the Live-Dead staining dye 96 h post-transfection and captured using the EVOS-M5000 microscope. Scale bar 300 µm. Data are represented as mean ± SEM in (b-c). ****p < 0.0001, ***p < 0.0005, *p < 0.05, ns: not significant.

To determine whether HOTPAM kinases promote mCRPC proliferation, we transfected C4-2B cells with individual siRNAs for HOXB13 as well as 8 HOTPAM kinase genes respectively (Fig. 3a,b) and examined impact on PC cell proliferation (Fig. 3c). Silencing either HOXB13 or HOTPAM9 kinase gene expression (*NCAPG*, *BUB1*, *NUF2*, *CIT*, *MKI67* and *AURKA*) significantly impacted C4-2B cell proliferation (Fig. 3c,d). These results reveal a novel network hijacked by HOXB13 to promote mCRPC growth and uncovers actionable targets in AR positive luminal epithelial subtype of PCs as these HOTPAM kinases are all potentially druggable entities.

### HOXB13 recruitment to HOTPAM9 metastasis signature genes is tumor-specific

**Figure 4:**
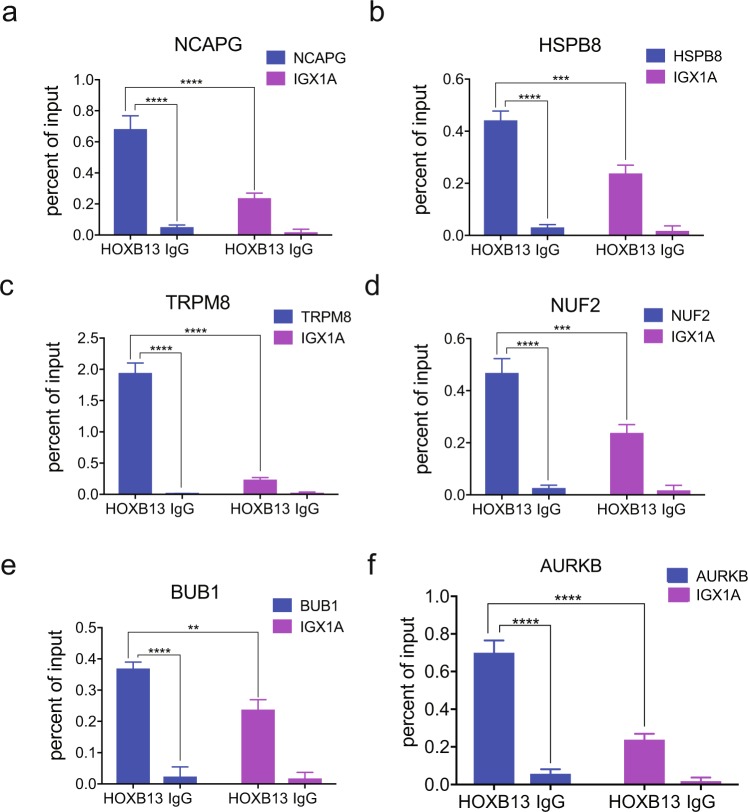
Recruitment of HOXB13 to a subset of HOTPAM9 target genes. (**a–g)** Directed ChIP-quantitative PCR of C4-2B chromatin extracts for the recruitment of HOXB13 to HOTPAM9 genomic regions including *NCAPG*, *HSPB8*, *TRPM8*, *NUF2*, *BUB1* binding sites. IGX1A is control site. *AURKB* is positive control gene for HOXB13 recruitment. Data are represented as mean ± SEM. ****p < 0.0001, ***p < 0.0005, **p < 0.01.

Distinct T-ARBS and HOXB13 peaks were found within a proximity of a subset of the HOTPAM9 metastasis signature genes (Supplementary Fig. 1b). ChIP sequencing data (GSE56288) revealed recruitment of AR to the vicinity of the HOTPAM9 genomic regions in the tumor tissues but not in normal prostates (Supplementary Fig. 1b). We synthesized primers corresponding to the AR/HOXB13 peak enrichment sites in the **Ch**romatin **I**mmunoprecipitation-sequencing (ChIP-sequencing-GSE56288) data of human prostate tumors^24^. Importantly, these sites were also enriched for HOXB13 binding motifs in HOXB13 ChIP sequencing data. Chromatin immunoprecipitation with HOXB13 antibody revealed binding at *NCAPG*, *TRPM8*, *NUF2*, *BUB1*, and *HSPB8* (Fig. 4a–f) loci suggesting recruitment of HOXB13 to these target genes. We have previously identified AURKB as a HOXB13 transcriptional target in prostate cancer.

### Clinical correlations of HOTPAM9 gene expression with survival

**Figure 5:**
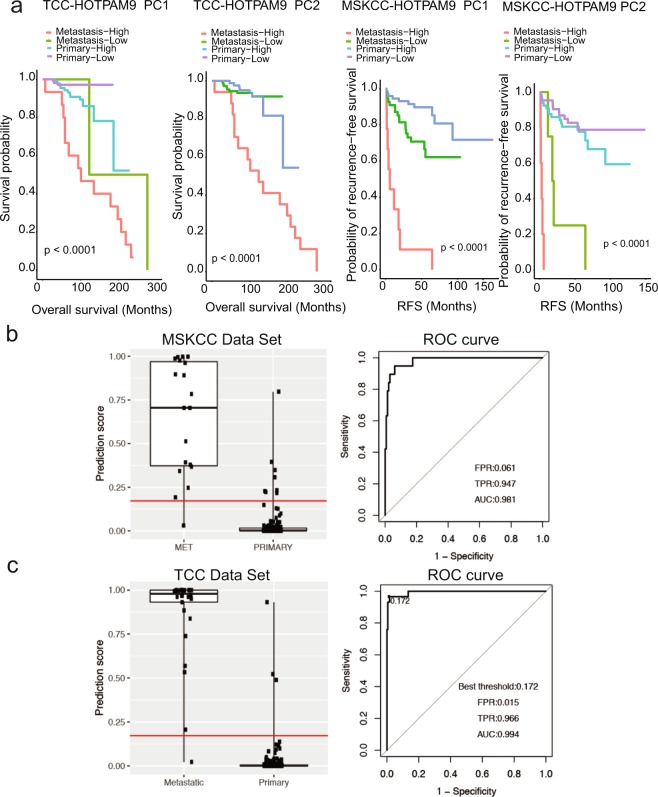
Kaplan-Meier survival analysis based on categorized gene expression levels of the HOTPAM9 genes. **(a)** Kaplan-Meier survival curves based on categorized gene expression levels for all the HOTPAM9 genes (n = 223). Overall survival stratified based on high or low gene expression analysis for the Moffitt TCC data set. For all the HOTPAM9 genes the overall log rank test is significant. *p* value between primary groups is shown in each plot (left two panels). Kaplan-Meier Recurrence-Free survival (RFS) curves are based on categorized gene expression levels for all the HOTPAM9 genes (n = 173). For RFS in the MSKCC data set, the overall log rank tests are all significant (right two panels). (**b)** Performance of statistical stratification model built on the basis of gene expression data of CIT/STK21 and HSPB8 as biomarkers to distinguish indolent from aggressive disease in MSKCC training data set. Stratification scores for the aggressive disease were compared between patients with primary tumors and those with metastatic tumors (left). ROC curve analysis was used to evaluate an ability to distinguish patients with primary cancer from those with metastatic disease (right). **(c)** Performance of the stratification model on Moffitt TCC data set by dividing patients’ classification scores at the optimal threshold (left). ROC curve was used to evaluate overall performance of the stratification model (right).

Subsequently we examined the association of *HOXB13* gene expression with multiple clinical parameters in the Moffitt TCC (Table 1) and MSKCC data sets respectively (Supplementary Table 1). Kaplan-Meier survival curves were generated based on categorized high and low gene expression levels for the HOXB13 genes with Moffitt and MSKCC data sets (Supplementary Fig. 4). Notably, the overall log rank test is significant for HOXB13 when considering overall survival for Moffitt TCC data set and recurrence free survival (RFS) for MSKCC data set. Further, in the Moffitt data set, based on log-rank test and univariate Cox proportional hazard regression analysis, only age was found to be significantly associated with overall survival time for patients with primary PC (Supplementary Fig. 5). In the MSKCC data set, Gleason score, clinical tumor stage, margin status and PSA at diagnosis were significantly associated with patients’ recurrence-free survival time (all log-rank test p-value < 0.05) (Supplementary Fig. 6). Kaplan-Meier survival probability curves were generated based on Principle Component 1(PC1) and 2 (PC2) with Moffitt and MSKCC data sets (Fig. 5). Notably, the overall log rank test is significant when considering overall survival for PC1 and PC2 in Moffitt TCC data set and recurrence free survival (RFS) for the MSKCC data set.

**Table 1:** Association between baseline clinical characteristics with patients’ disease type for Moffitt TCC data.

Variable	Metastatic	Primary	P-value
	N = 29	N = 194	
Age at diagnosis (years) <64 Years	8 (27.6%)	130 (67.0%)	0.014
> = 64 Year	13 (44.8%)	63 (32.5%)	
missing	8 (27.6%)	1 (0.5%)	
Gleason Score 5–6	—	64 (33.0%)	
7	—	125 (64.4%)	
8–10	—	3 (1.5%)	
Missing	—	2 (1.0%)	
Clinical Tumor Stage T1A,T1C	—	152 (78.4%)	
T2,T2A,T2B,T2C	—	37 (19.1%)	
Missing	—	5 (2.6%)	
Lymph node involvement			0.023
N1	1 (3.4%)	1 (0.5%)	
N0	1 (3.4%)	172 (88.6%)	
Missing	27 (93.1%)	21 (10.8%)	
Margin Status Negative	—	144 (74.2%)	
Positive	—	49 (25.3%)	
Missing	—	1 (0.52%)	
Hormone(second course)			<0.001
No	0 (0%)	186 (96.4%)	
Yes	5 (17.2%)	7 (3.6%)	
Missing	24 (82.8%)	1 (0.5%)	

^*^P-values for categorical variables calculated using the Fisher’s Exact method.

Kaplan-Meier overall survival analysis for the HOTPAM9 genes in the Moffitt TCC data set revealed low *NEK2* downregulation (↓;), *BUB1* (↓), and *NCAPG* (↓) were significantly associated with better overall survival times (Log-rank test p < 0.05). In contrast to these genes, high *HSPB8* (↑; over-expression) was observed to be significant (p = 0.0001) (Supplementary Fig. 7). In the MSKCC data set low *NEK2* (↓), *BUB1* (↓), and *NCAPG* (↓) were significantly associated with better RFS (Log-rank test p < 0.0001) while high *HSPB8* (↑) was significant (p = 0.0001) (Supplementary Fig. 8). Gleason score, clinical tumor stage, lymph node involvement, margin status, *NEK2*, *BUB1* and *NCAPG* in 9-genes cluster were significant by univariate Cox proportional hazard regression analysis. For multivariable analysis, Gleason score, lymph node involvement and *BUB1* in 9-genes cluster were significant (Hazard ratio for *BUB1* = 3.94 and 95% confidence interval = [1.58, 9.86]) (Table 2).

**Table 2:** Univariate and Multivariable Recurrence-Free Survival Analysis for Primary Patients in MSKCC data set.

Univariate Analysis					Overall Variable P Value	Multivariable Analysis		
Variable	Reference	Level	HR (95% CI)	Level Comparison P Value		HR (95% CI)	Level Comparison P Value	Overall Variable P Value
Age at diagnosis (years)	<64 Years	> = 64 Year	1.59 (0.69,3.65)	0.2748	0.2748			
Gleason Score	5–6	7	3.92 (0.89,17.25)	0.0709	<0.0001	2.37 (0.52, 10.78)	0.2627	<0.0001
	5–6	8–10	31.19 (6.83,142.36)	<0.0001		19.5 (4, 95.09)	0.0002	
Clinical Tumor Stage	T1C	T2,T2A,T2B,T2C	0.69 (0.29,1.63)	0.3994	0.0018			
	T1C	T3,T3A,T3B,T3C	6 (1.96,18.43)	0.0017				
Lymph node involvement	Normal_N0	Abnormal_N1	8.67 (3.2,23.46)	<0.0001	<0.0001	10.65 (3.18, 35.67)	0.0001	0.0001
Margin Status	Negative	Positive	2.7 (1.26,5.79)	0.0106	0.0106			
PSA at Diagnosis	<10	> = 10	2.25 (0.98,5.17)	0.0557	0.0557			
MKI67	Low	High	2.05 (0.95,4.43)	0.0663	0.0663			
NEK2	Low	High	2.59 (1.16,5.78)	0.0198	0.0198			
AURKA	Low	High	0.81 (0.37,1.74)	0.5810	0.5810			
BUB1	Low	High	3.02 (1.35,6.73)	0.0069	0.0069	3.94 (1.58, 9.86)	0.0034	0.0034
CIT	Low	High	1.62 (0.76,3.45)	0.2117	0.2117			
HSPB8	Low	High	0.5 (0.23,1.07)	0.0725	0.0725			
NCAPG	Low	High	2.54 (1.16,5.56)	0.0198	0.0198			
TRPM8	Low	High	1.52 (0.69,3.32)	0.2963	0.2963			
NUF2	Low	High	1.59 (0.75,3.41)	0.2294	0.2294			

Backwards Selection at removal alpha = 0.05.

### Identification of a two- gene subset, *CIT/STK21* and *HSPB8*, that facilitates stratification of metastatic PCs

As a further refinement of the core HOTPAM9 gene set, we built a multi-gene expression model to stratify primary from metastatic PC. The final output of the model is a probability score of developing metastases of PC. Each statistical model was trained on the basis of 150 patients in the MSKCC data set and came out with an optimal threshold value to classify patients with primary versus metastatic cancer. Receive operating characteristic (ROC) analysis showed an overall balanced accuracy of 98.8% and a significantly high area under the curve of 0.981 and a threshold value of 0.2347 in its evaluation of the MSKCC data set (Fig. 5b). The optimal model and threshold were then tested on Moffitt TCC data set to assess stratification performance of the model. Applying this model to the Moffitt TCC data set resulted in an overall balanced accuracy of 90.8% and a significantly high area under the curve (AUC) of 0.994 (p < 0.01), as compared to AUC of 0.5 for a random classification (Fig. 5c). Notably, our model revealed that two selective genes, the cell division gene *CIT/STK21* and the putative tumor suppressor gene *HSPB8* were sufficient to predict metastasis.

### HOXB13-CIT kinase axis predicts poor prognosis of metastatic prostate cancers

**Figure 6:**
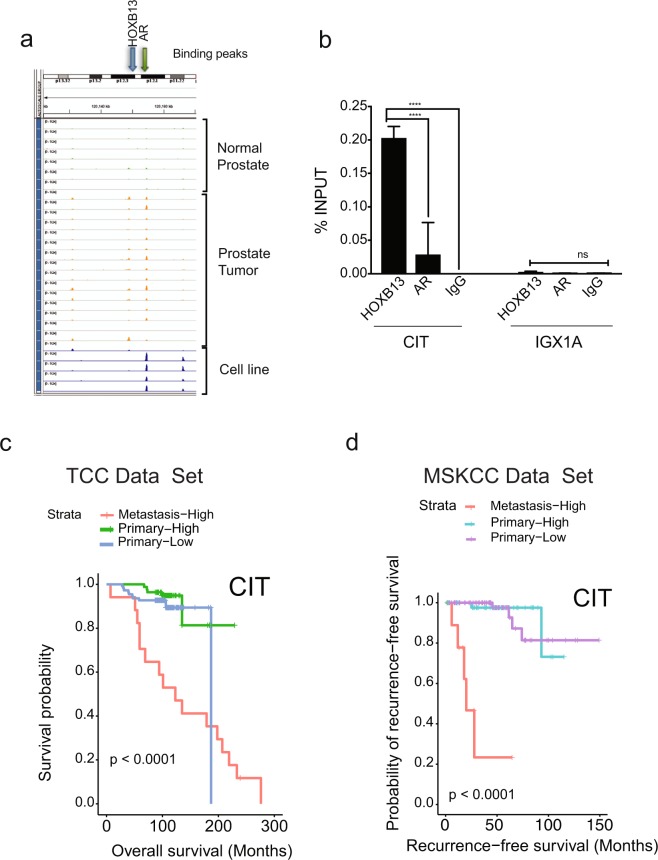
CIT/STK21 serine-threonine kinase is a direct target of HOXB13 in prostate cancer. **(a)** Analysis of HOXB13 or AR recruitment sites in the proximity of the CIT/STK21 in normal prostate (1–7 green peaks), human prostate tumors (8–20, orange peaks) and FOXA1 ChIP-seq in tumor (orange peak - line 21), HOXB13 ChIP-seq tumor (orange peak - line 22), HOXB13 ChIP-seq in LNCaP (blue peak - line 23), AR ChIP-seq in normal prostate cell line LHSAR transfected with HOXB13, FOXA1, HOXB13 plus FOXA1 (blue peaks- line 24–27) (ChIP-seq data from GSE56288). **(b)** Directed ChIP-quantitative PCR for the recruitment of HOXB13 to CIT/STK21 promoter with anti-HOXB13 antibodies, anti-AR antibodies or IgG (control). IGX1A is control site. ± SEM. ****p < 0.0001, ns: not significant. **(c**,**d)** Kaplan-Meier overall survival curves based on categorized gene expression levels of HOTPAM9 genes in Moffitt TCC data set and Recurrence Free Survival curves in the MSKCC data set. *p* values were generated by the log-rank test.

As reduction in both CIT kinase and HOXB13 negatively impacted mCRPC proliferation, we examined whether it is a direct transcriptional target of HOXB13. Analysis of ChIP sequencing data (GSE56288) for the recruitment of HOXB13 and AR to the vicinity of the *CIT/STK21* revealed overlapping peaks, consistent with our original selection for tumor-specific ARBS. Directed ChIP-quantitative PCR revealed binding of HOXB13 in the proximity of the *CIT* promoter but not at a control site, *IGX1A* (Fig. 6a,b). Conversely, high expression of CIT kinase in metastatic PCs is associated with poor outcomes in both Moffitt and MSKCC data sets (Fig. 6c,d). Collectively, our results indicate CIT kinase as a potential biomarker and therapeutic vulnerability in metastatic PCs.

### HSPB8 mRNA levels are inversely correlated with HOXB13 and AR expression in metastatic PCs

**Figure 7:**
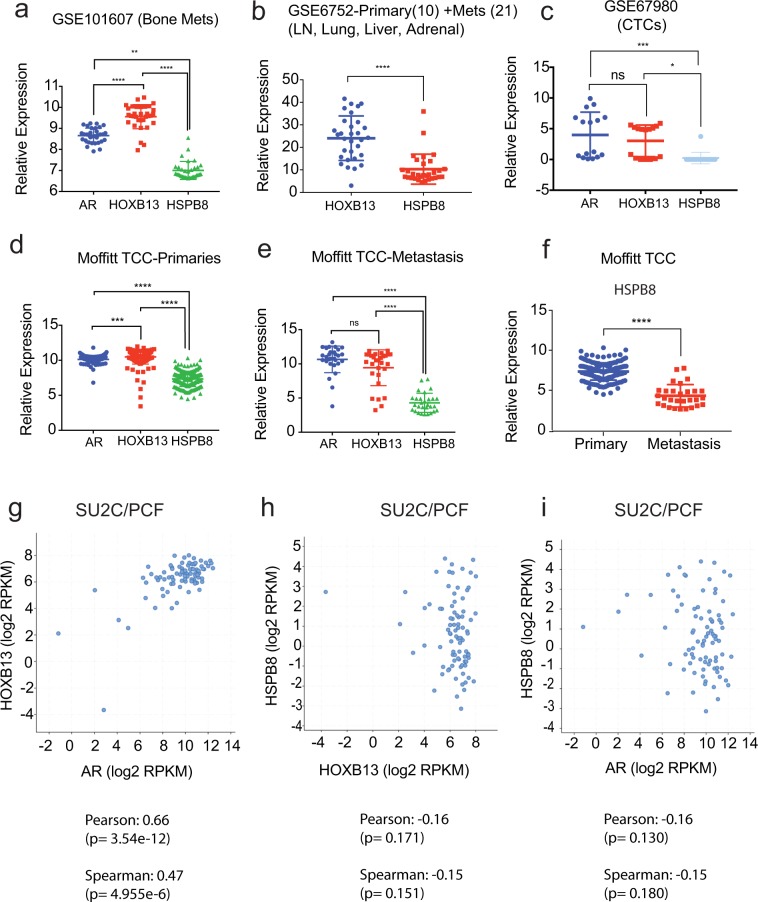
HSPB8 expression is downregulated in metastatic prostate cancer. **(a)** Comparison of AR, HOXB13, and HSPB8 gene expression in bone metastatic CRPCs (GSE101607) **p < 0.005, ****p < 0.0005. **(b)** GSE6752 which comprised 10 primary and 21 androgen ablation resistant metastatic samples. ****p < 0.0005. **(c)** GSE67980 (human primary and metastatic cases) ***p < 0.005; *p < 0.05. ns = not significant. **(d)** Moffitt TCC primaries. ****p < 0.0005; *** p < 0.005. **(e)** Moffitt TCC metastasis. ****p < 0.0005. **(f)** Moffitt TCC Data Set. ****p < 0.0005. **(g)** AR/HOXB13 co-expression is observed in 94 (63%) of metastatic PC cases (SU2C/PCF cohort, *Cell* 2015). HSPB8 expression inversely correlates with AR and HOXB13 expression. Pearson co-relation co-efficient and Spearman rank co-efficient are shown below each panel.

HOXB13 and AR are co-expressed in metastasis at distant sites and positively correlate in multiple clinical data sets (Fig. 7a–c; Supplementary Fig. 9). Conversely, we observed that HSPB8 mRNA levels showed inverse correlation with HOXB13 and AR mRNA expression  in multiple gene expression data sets including Moffitt TCC data set, GSE21034, GSE101607, GSE6752, GSE67980 and SU2C/PCF datasets (Fig. 7a–i; Supplementary Fig. 9). Even when comparing primary to metastasis within the Moffitt TCC data set as well as in the GSE6919 dataset (196 primary and 75 metastatic samples), *HSPB8* mRNA levels were significantly lower, (log2 fold change −1.952; *p* value = 6.76E-15) (Fig. 7f; Supplementary Fig. 2d, Supporting Data Table 10). In contrast, HSPB8 mRNA expression was significantly upregulated in multiple metastasis cell line models following HOXB13 reduction (Supplementary Fig. 2e). Both castration-sensitive and castration-resistant metastatic PCs showed similar levels of expression of *AR*, *HOXB13* and the HOTPAM genes (Supplementary Fig. 10).

### Effect of HSPB8 overexpression on PC cell migration

**Figure 8:**
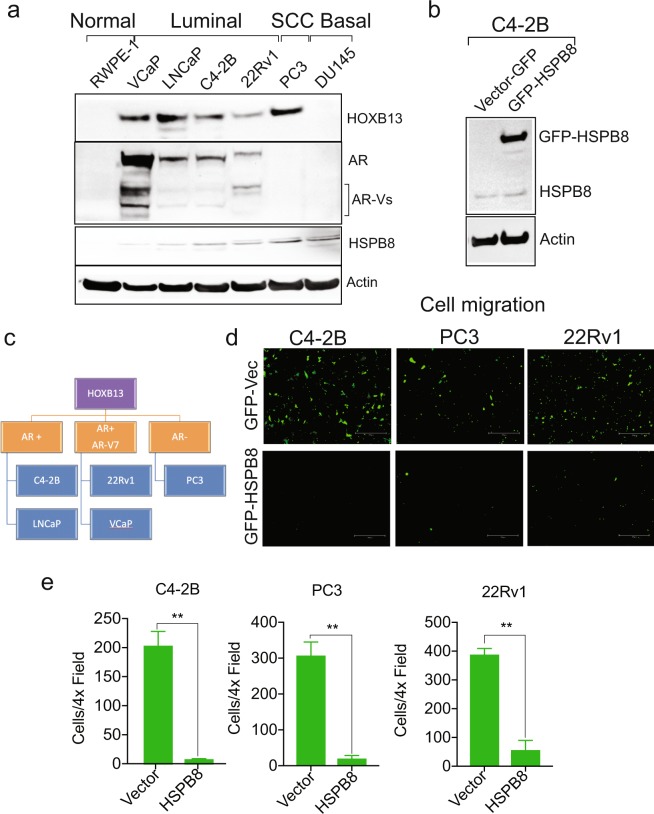
Overexpression of HSPB8 inhibits migration of metastatic prostate cancer cells. **(a)** Expression of HOXB13, AR and HSPB8 in various cell line models. RWPE-1 is AR-low; VCaP, LNCaP, C4-2B and 22Rv1 are AR-positive; PC3 and DU145 are AR-negative cell lines. **(b)** C4-2B cells were transfected with 4 µg of GFP-Vector or GFP-HSPB8. Whole cell lysates were immunoblotted with anti-HSPB8 antibody. Actin is a normalization control. **(c)** Stratification of PC cell lines based on their HOXB13, AR and AR-V7 expression. **(d**,**e)** C4-2B, PC3 and 22Rv1 cells were transfected with 4 µg of GFP-Vector or GFP-HSPB8. Transwell cell migration assay was performed using fluroblock inserts. **(d)** After 16 h, cells were visualized and captured using the EVOS-M5000 microscope. Scale bar 300 µm. **(e)** Quantitative analysis of GFP-positive migrated cells. n = 2 independent replicates. ± SEM. **p < 0.01.

Immunoblot analysis revealed that HOXB13 is expressed in AR positive PC cell lines of luminal epithelial origin as well as in AR negative PC3, a cancer of small cell origin (Fig. 8a). We observed that HSPB8 protein is expressed at very low levels in multiple PC cell lines tested (Fig. 8a,b). To determine whether HOXB13 mediated HSPB8 downregulation is a mechanism to facilitate PC cell migration, C4-2B were transfected with GFP vector or GFP-HSPB8 (Fig. 8b). Further, the effect of HSPB8 overexpression was also examined in AR-V7 positive 22Rv1 and AR negative PC3 cells (Fig. 8c). Overexpression of HSPB8 significantly reduced the migration of highly aggressive PC cells (Fig. 8d,e).

### The tumor suppressor gene HSPB8 is a target of HOXB13

**Figure 9:**
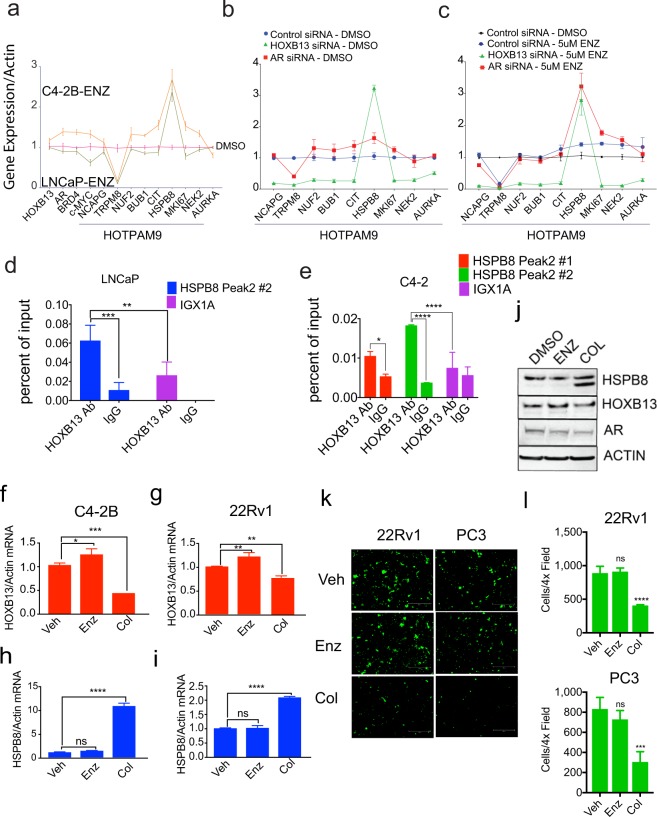
Colchicine a microtubule inhibitor induces HSPB8 protein expression and inhibits mCRPC migration. **(a)** Androgen-responsive LNCaP and androgen-independent C4-2B cells were treated with Vehicle (DMSO) or 5 µM of Enzalutamide. Total RNA was isolated, followed by qRT-PCR with HOXB13, c-MYC and HOTPAM9 primers. **(b,c)** C4-2B cells were transfected with control, HOXB13 or AR siRNAs, followed by treatment with Vehicle or Enzalutamide (5 µM) and expression of HOTPAM9 genes was determined by qRT-PCR. **(d,e)** Directed ChIP-quantitative PCR for HOXB13 recruitment to sites upstream of the *HSPB8* gene with HOXB13 antibody or IgG in LNCaP **(d)** and C4-2B **(e)** cells. Chromosomal locations (Peak1: Chr:119,614,158- 119,614,438) and (Peak2: Chr:119,616,400- 119,616,900). Data are represented as mean ± SEM. ****p < 0.0001; ***p < 0.001; **p = 0. 0025, *p < 0.05t. C4-2B and 22Rv1 were treated with Vehicle (DMSO), 10 µM of Enzalutamide (Enz) or 1 µM of Colchicine (Col) for 24 h. **(f-i)** Total RNA was isolated, followed by qRT-PCR with HOXB13, HSPB8 and Actin primers. **(j)** Whole cell lysates were immunoblotted with anti-HSPB8, anti-HOXB13 and anti-AR antibody. Actin is a normalization control. **(k)** Transwell cell migration assay was performed on GFP-positive PC3 and 22Rv1 cells using fluroblock inserts. Scale bar 300 µm. n = 2 independent replicates. (**l**) Quantitation of cell migration shown in (k). Data are represented as mean ± SEM in (f-i and l). ****p < 0.0001, ***p < 0.0005, **p < 0.01, *p < 0.05, ns: not significant.

To confirm the regulation of the HOTPAM9 genes by *HOXB13* and the effect of anti-androgens, we analyzed their expression in androgen-responsive (LNCaP) and bone metastatic castration-resistant model cell line (C4-2B) treated with vehicle and Enzalutamide. A majority of the HOTPAM9 mRNA levels were expressed at higher levels in C4-2B treated with Enzalutamide compared to LNCaP except for *TRPM8* whose levels decreased, indicating an androgen regulated gene. In contrast, *HSPB8* levels increased significantly in response to Enzalutamide (Fig. 9a). To ascertain *HOXB13* and/or *AR* dependency of the HOTPAM9 gene expression, C4-2B cells were transfected with Control, *HOXB13* or *AR* silencing RNAs, and the expression of HOTPAM9 genes were analyzed in the vehicle and Enzalutamide treated cells (Fig. 9b,c). In HOXB13 siRNA transfected C4-2B cells, *HSPB8* expression was significantly increased while *AR* silencing had a marginal effect (Fig. 9b). However, in cells treated with Enzalutamide, depletion of either HOXB13 or AR mRNA led to a significant upregulation of *HSPB8* gene expression compared to Control (Fig. 9b,c). ChIP-sequencing analysis revealed two HOXB13 binding sites upstream of *HSPB8 * (Fig. 9d,e).

Colchicine, a tubulin inhibitor, has been reported to induce expression of *HSPB8* as well as inhibit PC growth^32,33^. While we did not detect accumulation of HSPB8 protein levels in response to Enzalutamide in C4-2B, we observed that treatment with Colchicine decreased HOXB13 and also induced HSPB8 mRNA and protein expression (Fig. 9f–j). Moreover, consistent with our overexpression studies, treatment of PC cells with Colchicine but not Enzalutamide impaired migration of PC cells (Fig. 9k,l).

## Discussion

**Figure 10:**
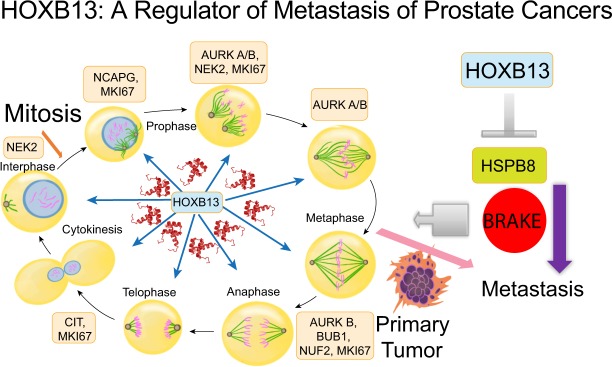
A schematic showing key mitotic kinases over-expressed in metastatic PCs and regulated by HOXB13. HSPB8 functions as a restraint against PC metastasis.

Our present study suggests that HOXB13, a critical regulator of metastatic PC growth^16,34,35^, directs a robust pro-proliferative androgen-independent transcriptional program by increasing the expression of a subset of mitotic kinases (Fig. 10: Model). Indeed, Wang *et*. *al*., reported upregulation of mitotic cell cycle genes in PC cells growing under long term conditions of androgen deprivation as well as in clinical androgen independent PC samples^36^. Increased expression of cell cycle kinases such as AURKA, BUB1, BUB3 and PIM1 is frequently observed in metastatic PCs^5,8,10,37,38^. Further, extensive molecular profiling comparing primary and metastatic human PCs has yielded significant insights into the different subsets as well as actionable targets of prognostic significance in metastatic PCs^4,6,8,10,37–41^. In agreement with these studies, gene ontology (GO) studies revealed that the top ten pathways affected as a result of *HOXB13* knockdown were associated with the cell cycle, including DNA replication, G1/S phase, and the metaphase checkpoint. Moreover, that complete deletion of *HOXB13* in LNCaP, VCaP and C4-2B PC models is lethal is consistent with its essential role in directing the expression and function of mitotic checkpoint kinases. We did not observe a significant enrichment of HOXB13 target gene signature in NPCRPC vs NP or NPp53, suggesting some degree of mutual exclusivity of HOXTAR genes with PTEN and p53 deletion. As PTEN and TP53 (as well as RB1) aberrations are a frequent genetic aberration in advanced/ metastatic prostate cancers^4,10,38^; HOXB13 in metastasis/CRPCs with no aberrations in TP53/ PTEN, could define a new subset.

Alterations in expression of a subset of these HOTPAM9 mitotic kinases have previously been reported to potentiate tumorigenesis through increased genome instability and aneuploidy^42–46^. For example, overexpression of *NEK2*, encoding a serine/threonine kinase associated with centrosome duplication and spindle assembly checkpoint, has been found in various malignancies and linked to poor prognosis^42,47^. While the 2 -geneset is sufficient for metastasis prediction, the HOTPAM9 gene set is more informative into kinases that are specifically overexpressed in metastatic populations that likely facilitate rapid proliferation and clonal expansion than their primary counterparts. Indeed, the overexpression of mitotic kinases is consistent with a role for centrosomal amplification and mitotic propensity in driving intratumoral heterogeneity, tumor progression and metastasis^48^.

CIT/STK21 kinase is a direct transcriptional target of *HOXB13* and an increase in *CIT* gene expression in conjunction with a decrease in *HSPB8* expression is sufficient to indicate propensity for metastasis. A tumor-promoting role for CIT kinase has also been proposed for other cancers such as colorectal, breast, and cervical cancers^43,49,50^. While potential interacting partners and substrates of CIT kinase have been identified, the mechanism by which *CIT/STK21* expression is regulated in cancers in general and PC in particular is not known^51^. Reduced expression of CIT is associated with defective cytokinesis^51^, and in our study loss of *CIT* or *HOXB1*3 impacted cell proliferation suggesting a dependency of PC cells on HOXB13-CIT transcriptional axis for survival. STRING analysis revealed enrichment of mitotic kinases in the HOTPAM9 genes uncovered *CIT* kinase, not, linked to other genes. This is a hitherto uncharacterized association that we have identified through this study. In PC3 cells, c-MYC expression was induced following HOXB13 knockdown suggesting a functional redundancy in AR negative cells for regulating expression of mitotic kinases. However, AR positive cancers appear to rely more predominantly on HOXB13 for proliferation.

Activation of oncogenes that relay mitogenic signals and inactivation of tumor suppressors that restrain growth is a hallmark of many cancers. A striking observation in this study is the consistent downregulation of *HSPB8* gene expression to a certain extent in primary but quite significantly in metastatic PCs. *HSPB8*, codes for a 22 kDa heat shock protein, has been shown to clear mutant AR protein in motoneuronal cells^52^. We observed that the gene expression of *HSPB8*, an atypical member of the heat shock protein family, inversely correlated with *AR* and *HOXB13* mRNA expression in metastatic PCs from multiple organ sites, particularly bone metastatic PCs (Supplementary Fig. 9; Supporting Data Tables 11–13). Notably, treatment with AR antagonist Enzalutamide did not suppress HOXB13 mRNA expression and despite an increase in HSPB8 mRNA it was not sufficient to induce HSPB8 protein expression. In contrast, in Colchicine-treated cells, HOXB13 was not only downregulated but this also led to a significant restoration of HSPB8 protein levels and a significant inhibition of cell migration. This is consistent with recent studies that demonstrated that overexpression of HSPB8 led to cell cycle arrest and apoptosis in certain melanoma models^29,53^. It remains to be seen whether HSPB8 is the main target of Colchicine action and whether it can be employed to prevent metastatic progression of *HOXB13* positive PCs in pre-clinical mouse models.

These results with Enzalutamide suggest that the androgen-bound AR may either inhibit HOXB13 from binding to the *HSPB8* gene locus in primary tumors or it may sequester HOXB13 from a repressive effect on *HSPB8* gene expression. A suppressive effect of HOXB13 on androgen-stimulated PSA expression by AR has been proposed as mechanism of regulation in a context dependent manner^11,21^. Androgen depletion may facilitate HOXB13 recruitment to the *HSPB8* gene locus to suppress its expression and override the brake on cell proliferation. In corollary, a pro-proliferative role for HSPB8 has been reported in certain breast cancer models. In these studies, it was observed that HSPB8 protein is induced in response to estrogen stimulation of MCF7 and T47D (estrogen-dependent cell lines) breast cancer cells, whereas it was neither detected nor induced in the triple negative highly invasive MDA-MB231 breast cancer cell lines; overexpression caused a modest accumulation of cells in G2/M^54^. Further, *HSPB8* is linked to tamoxifen resistance in breast cancers and correlates with poor clinical outcome^55^. Collectively these results suggest a context dependent role for HSPB8 in hormone refractory cancers.

An area of future investigation is whether HOXB13 actively recruits co-repressors at the *HSPB8* genomic locus to suppress its expression in metastatic PC cells. *HSPB8* is expressed at low levels in certain PC models and silenced by methylation^56^. Which histone methyltransferase or DNA methyltransferase act in conjunction with HOXB13 to repress HSPB8 expression during metastatic progression remains to be determined. Overall, our study reveals HOXB13 as a master transcriptional regulator of PC metastasis and has identified actionable targets for the treatment of HOXB13 overexpressing primary and metastatic prostate cancers.

## Materials and Methods

### Cell culture, antibodies and vectors

RWPE-1, PC3, DU145, VCaP, 22Rv1 and LNCaP were obtained from American Type Culture Collection (ATCC), that are authenticated by Short Tandem Repeat Profiling and used within 5–10 passages and replenished from stocks. C4-2B is described earlier^2^. All cell lines were incubated in a humidified atmosphere of 5% CO_2_ at 37 °C and cultured as described earlier^2^. Cultures are routinely tested for Mycoplasma contamination using the MycoAlert™ PLUS Mycoplasma Detection Kit from Lonza and by PCR based screening. HOXB13 F-9 monoclonal antibody (Cat #SC-28333) was purchased from Santa Cruz and HOXB13 rabbit polyclonal antibodies were purchased from Genetex (GTX129245). HSPB8 (Abcam Cat# Ab79784), Actin antibodies (Sigma-Aldrich Cat# A2228), were purchased from Abcam. AR (F39.4.1) antibody was purchased from Biogenex. siRNAs were purchased from multiple sources. Source for all siRNA and sequences are provided in the Supporting data section. GFP-HSPB8 expression vector was synthesized and sequenced by Genscript. Prostate cancer cells were transfected with siRNAs or plasmids using LONZA nucleofector kit.

### Gene expression profiling

Total RNA was extracted from the prostate cells (QIAGEN mRNAeasy kit, Germany) and analyzed as described earlier^16^. Primers used for qRT-PCR for detection of HOTPAM9 genes, *HOXB13*, *AR*, AR-V7, PSA (*KLK3*), *NKX3.1*, *TMPRSS2*, *c-MYC* and Actin, as well as primers for ChIP analysis of *HSPB8*, *CIT, AURKB, NCAPG, TRPM8, NUF2 * and *IGX1A* (control primers) are listed in Supporting data section.

### RNA sequencing data analysis

Heat maps and Venn diagrams were produced with Partek (Partek, Inc.) and GENE-E software from Broad Institute (https://software.broadinstitute.org/GENE-E/). Analysis of overlaps from the lists of differentially expressed genes was performed using custom Perl scripts.

### Moffitt total cancer care (TCC) data set

Moffitt Information Shared Services/Collaborative Data Services Core provided the de-identified gene expression data for the 194 primary and 29 metastatic tumors (Rosetta/Merck Human RSTA Custom Affymetrix 2.0 microarray [HuRSTA_2a520709.CDF]) from the Moffitt Total Cancer Care (TCC) dataset. Studies that fall under the TCC protocol have been obtained with patients’ written consent.

### External validation data set

We downloaded GSE21034 series, (MSKCC dataset)^28^ and GSE6919^57^ from Gene Expression Ominibus (GEO) for external validation. The MSKCC data set for bioinformatics analysis comprised 179 samples (29 normal, 131 primary and 19 metastatic tumors). The GSE6919 dataset comprised 504 samples (233 normal, 196 primary and 75 metastatic tumors) The downloaded raw data were normalized using quantile normalization. The heat map of each gene set was generated using heatmap2 with in-house R scripts^16^.

### Principle component analysis (PCA)

Gene expression data for the HOXTAR87 genes were extracted from the Moffitt TCC data set as well as the MSKCC data set (Supporting data section). PCA was performed using the Evince software (Prediktera, ver2.7.9). We observed a separation between primary and metastatic samples in the second component. To identify a minimal gene set driving this separation, we extract the PCA loading values of each gene. We selected the genes with very highest/smallest loading values. The genes with smallest absolute loading values were gradually removed from the list so that the first component value is not reduced. Finally, nine gene sets were obtained which can separate the primary from the metastatic with the highest first component value of 58%.

### Cell proliferation and cell migration assay

Cell number was determined by harvesting cells, staining with Trypan blue and counting by hemocytometer. Cell migration was performed using Fluroblock transwell cell migration assay.

### Statistical analysis

Differences in means between individual groups were analyzed by Student’s *t*-test, or analysis of variance (ANOVA). Two-sided p-values < 0.05 were considered statistically significant.

### Statistical analysis for human tumor data

Descriptive analysis was conducted for all the variables interested (age, Gleason score, clinical tumor stage, lymph node involvement, and margin status, PSA at diagnosis and gene expression data for selected genes). Fisher’s exact test was used to compare distribution of clinical variables between patients with primary cancer and those with metastatic cancer. Kaplan-Meier (KM) survival curves were plotted with R 3.3.2. For primary cancers, univariate and multivariable Cox proportional hazard regression analysis were used. Variables with two-sided *p-value* less than 0.05 in the univariate analysis were included in the multivariable analysis. Backward selection allowing variable removal at the significance level of 0.05 was used to screen significant variables. These statistical analyses were made using SAS 9.4. A multivariable logistic regression model was trained to predict metastatic PC patients on the basis of normalized gene expression of MSKCC data set. A stepwise variable selection method based on Akaike information criterion (AIC) was used to select the optimal logistic regression model. Receive operating characteristic (ROC) curve was used to evaluate overall performance of the logistic regression model in classifying primary patients from those with metastatic disease^58^. Wilcoxon rank sum test was used to compare Gleason scores of two groups. These statistical analyses were performed using R 3.3.2.

## Supplementary information

Supplementary Figures

Supplementary data set

## Data Availability

Materials, gene expression data and associated protocols will be available upon reasonable request.

## References

[1] Kim EH, Andriole GL (2018). Prostate Cancer Review. Mo Med.

[2] Mahajan K (2017). ACK1/TNK2 Regulates Histone H4 Tyr88-phosphorylation and AR Gene Expression in Castration-Resistant Prostate Cancer. Cancer Cell.

[3] Zou M (2017). Transdifferentiation as a Mechanism of Treatment Resistance in a Mouse Model of Castration-Resistant Prostate Cancer. Cancer Discov.

[4] Grasso CS (2012). The mutational landscape of lethal castration-resistant prostate cancer. Nature.

[5] Beltran H (2016). Divergent clonal evolution of castration-resistant neuroendocrine prostate cancer. Nat Med.

[6] Beltran, H., Antonarakis, E. S., Morris, M. J. & Attard, G. Emerging Molecular Biomarkers in Advanced Prostate Cancer: Translation to the Clinic. *Am Soc Clin Oncol Educ Book* **35**, 131–141, doi:10.14694/EDBK_159248 10.1200/EDBK_159248 (2016). 10.1200/EDBK_159248 27249694

[7] Park JW (2018). Reprogramming normal human epithelial tissues to a common, lethal neuroendocrine cancer lineage. Science.

[8] Chen F, Zhang Y, Varambally S, Creighton CJ (2019). Molecular Correlates of Metastasis by Systematic Pan-Cancer Analysis Across The Cancer Genome Atlas. Mol Cancer Res.

[9] Varambally S (2013). Prostate cancer genomics: progress and promise. Eur Urol.

[10] Varambally S (2005). Integrative genomic and proteomic analysis of prostate cancer reveals signatures of metastatic progression. Cancer Cell.

[11] Norris JD (2009). The homeodomain protein HOXB13 regulates the cellular response to androgens. Mol Cell.

[12] Chen Z (2018). Diverse AR-V7 cistromes in castration-resistant prostate cancer are governed by HoxB13. Proc Natl Acad Sci USA.

[13] Sharp Adam, Coleman Ilsa, Yuan Wei, Sprenger Cynthia, Dolling David, Rodrigues Daniel Nava, Russo Joshua W., Figueiredo Ines, Bertan Claudia, Seed George, Riisnaes Ruth, Uo Takuma, Neeb Antje, Welti Jonathan, Morrissey Colm, Carreira Suzanne, Luo Jun, Nelson Peter S., Balk Steven P., True Lawrence D., de Bono Johann S., Plymate Stephen R. (2018). Androgen receptor splice variant-7 expression emerges with castration resistance in prostate cancer. Journal of Clinical Investigation.

[14] Mallo M, Alonso CR (2013). The regulation of Hox gene expression during animal development. Development.

[15] Shah N, Sukumar S (2010). The Hox genes and their roles in oncogenesis. Nature reviews. Cancer.

[16] Nerlakanti N (2018). Targeting the BRD4-HOXB13 Coregulated Transcriptional Networks with Bromodomain-Kinase Inhibitors to Suppress Metastatic Castration-Resistant Prostate Cancer. Mol Cancer Ther.

[17] Beebe-Dimmer JL (2015). The HOXB13 G84E Mutation Is Associated with an Increased Risk for Prostate Cancer and Other Malignancies. Cancer Epidemiol Biomarkers Prev.

[18] Economides KD, Capecchi MR (2003). Hoxb13 is required for normal differentiation and secretory function of the ventral prostate. Development.

[19] Sreenath T, Orosz A, Fujita K, Bieberich CJ (1999). Androgen-independent expression of hoxb-13 in the mouse prostate. Prostate.

[20] Jung C, Kim RS, Zhang HJ, Lee SJ, Jeng MH (2004). HOXB13 induces growth suppression of prostate cancer cells as a repressor of hormone-activated androgen receptor signaling. Cancer Res.

[21] Kim SD (2010). HOXB13 is co-localized with androgen receptor to suppress androgen-stimulated prostate-specific antigen expression. Anat Cell Biol.

[22] Kim YR (2010). HOXB13 promotes androgen independent growth of LNCaP prostate cancer cells by the activation of E2F signaling. Mol Cancer.

[23] Kim YR (2014). HOXB13 downregulates intracellular zinc and increases NF-kappaB signaling to promote prostate cancer metastasis. Oncogene.

[24] Pomerantz MM (2015). The androgen receptor cistrome is extensively reprogrammed in human prostate tumorigenesis. Nat Genet.

[25] Edwards S (2005). Expression analysis onto microarrays of randomly selected cDNA clones highlights HOXB13 as a marker of human prostate cancer. British journal of cancer.

[26] Ylitalo Erik Bovinder, Thysell Elin, Jernberg Emma, Lundholm Marie, Crnalic Sead, Egevad Lars, Stattin Pär, Widmark Anders, Bergh Anders, Wikström Pernilla (2017). Subgroups of Castration-resistant Prostate Cancer Bone Metastases Defined Through an Inverse Relationship Between Androgen Receptor Activity and Immune Response. European Urology.

[27] Cardoso M, Maia S, Paulo P, Teixeira MR (2016). Oncogenic mechanisms of HOXB13 missense mutations in prostate carcinogenesis. Oncoscience.

[28] Taylor BS (2010). Integrative genomic profiling of human prostate cancer. Cancer cell.

[29] Smith CC (2012). Restored expression of the atypical heat shock protein H11/HspB8 inhibits the growth of genetically diverse melanoma tumors through activation of novel TAK1-dependent death pathways. Cell Death Dis.

[30] Liu T (2016). Anti-tumor activity of the TRPM8 inhibitor BCTC in prostate cancer DU145 cells. Oncology letters.

[31] Peng M (2015). Overexpression of short TRPM8 variant alpha promotes cell migration and invasion, and decreases starvation-induced apoptosis in prostate cancer LNCaP cells. Oncology letters.

[32] Crippa V (2016). Transcriptional induction of the heat shock protein B8 mediates the clearance of misfolded proteins responsible for motor neuron diseases. Sci Rep.

[33] Fakih M, Yagoda A, Replogle T, Lehr JE, Pienta KJ (1995). Inhibition of prostate cancer growth by estramustine and colchicine. Prostate.

[34] Barresi V, Ieni A, Reggiani Bonetti L, Tuccari G (2016). HOXB13 expression in metastatic prostate cancer. Virchows Arch.

[35] Barresi V (2016). HOXB13 as an immunohistochemical marker of prostatic origin in metastatic tumors. APMIS.

[36] Wang Q (2009). Androgen receptor regulates a distinct transcription program in androgen-independent prostate cancer. Cell.

[37] Singhal U (2018). Multigene Profiling of CTCs in mCRPC Identifies a Clinically Relevant Prognostic Signature. Mol Cancer Res.

[38] Dhanasekaran SM (2001). Delineation of prognostic biomarkers in prostate cancer. Nature.

[39] Pritchard CC (2016). Inherited DNA-Repair Gene Mutations in Men with Metastatic Prostate Cancer. N Engl J Med.

[40] Smith SC (2014). HOXB13 G84E-related familial prostate cancers: a clinical, histologic, and molecular survey. Am J Surg Pathol.

[41] Asangani IA (2014). Therapeutic targeting of BET bromodomain proteins in castration-resistant prostate cancer. Nature.

[42] Liu Q, Hirohashi Y, Du X, Greene MI, Wang Q (2010). Nek2 targets the mitotic checkpoint proteins Mad2 and Cdc20: a mechanism for aneuploidy in cancer. Experimental and molecular pathology.

[43] McKenzie Callum, Bassi Zuni I., Debski Janusz, Gottardo Marco, Callaini Giuliano, Dadlez Michal, D'Avino Pier Paolo (2016). Cross-regulation between Aurora B and Citron kinase controls midbody architecture in cytokinesis. Open Biology.

[44] Zeng YR (2015). Overexpression of NIMA-related kinase 2 is associated with progression and poor prognosis of prostate cancer. BMC urology.

[45] Marumoto T (2003). Aurora-A kinase maintains the fidelity of early and late mitotic events in HeLa cells. J Biol Chem.

[46] Tang Z, Sun Y, Harley SE, Zou H, Yu H (2004). Human Bub1 protects centromeric sister-chromatid cohesion through Shugoshin during mitosis. Proc Natl Acad Sci USA.

[47] Hayward DG, Fry AM (2006). Nek2 kinase in chromosome instability and cancer. Cancer Lett.

[48] Rida PC, Cantuaria G, Reid MD, Kucuk O, Aneja R (2015). How to be good at being bad: centrosome amplification and mitotic propensity drive intratumoral heterogeneity. Cancer Metastasis Rev.

[49] Wu Z (2017). Up-regulation of CIT promotes the growth of colon cancer cells. Oncotarget.

[50] McKenzie C, D’Avino PP (2016). Investigating cytokinesis failure as a strategy in cancer therapy. Oncotarget.

[51] Whitworth H (2012). Identification of kinases regulating prostate cancer cell growth using an RNAi phenotypic screen. PLoS One.

[52] Rusmini P (2013). Clearance of the mutant androgen receptor in motoneuronal models of spinal and bulbar muscular atrophy. Neurobiol Aging.

[53] Li B (2007). Overload of the heat-shock protein H11/HspB8 triggers melanoma cell apoptosis through activation of transforming growth factor-beta-activated kinase 1. Oncogene.

[54] Piccolella M (2017). The small heat shock protein B8 (HSPB8) modulates proliferation and migration of breast cancer cells. Oncotarget.

[55] Gonzalez-Malerva L (2011). High-throughput ectopic expression screen for tamoxifen resistance identifies an atypical kinase that blocks autophagy. Proceedings of the National Academy of Sciences of the United States of America.

[56] Gober MD, Smith CC, Ueda K, Toretsky JA (2003). & Aurelian, L. Forced expression of the H11 heat shock protein can be regulated by DNA methylation and trigger apoptosis in human cells. The Journal of biological chemistry.

[57] Chandran UR (2007). Gene expression profiles of prostate cancer reveal involvement of multiple molecular pathways in the metastatic process. BMC Cancer.

[58] Perkins NJ, Schisterman EF (2006). The inconsistency of “optimal” cutpoints obtained using two criteria based on the receiver operating characteristic curve. Am J Epidemiol.

