# Supplementary material for: The Homeobox gene, HOXB13, Regulates a Mitotic Protein-Kinase Interaction Network in Metastatic Prostate Cancers
Source: Sci Rep. 2019 Jul 4;9:9715. doi: 10.1038/s41598-019-46064-4 (PMC6609629; doi:10.1038/s41598-019-46064-4)
Supplement: Supplementary file 1 — Supplementary Figures [file 41598_2019_46064_MOESM1_ESM.pdf]

Supplementary Figures

The Homeobox gene, *HOXB13*, Regulates a Mitotic Protein-Kinase Interaction Network in Metastatic Prostate Cancers

Jiqiang Yao[1*], Yunyun Chen[1*], Duy T. Nguyen[2*], Zachary J. Thompson[1], Alexey M. Eroshkin[3], Niveditha Nerlakanti[4], Ami K. Patel[4], Neha Agarwal[4], Jamie K. Teer[1], Jasreman Dhillon[5], Domenico Coppola[5], Jingsong Zhang[6], Ranjan Perera[7], Youngchul Kim[1], and Kiran Mahajan[2#]

[1]Department of Biostatistics and Bioinformatics, H. Lee Moffitt Cancer Center and Research Institute, 12902 Magnolia Drive, Tampa, FL.

[2]Department of Surgery, Washington University in St. Louis, MO, USA.

[3]Bioinformatics Core, Sanford Burnham Prebys Medical Discovery Institute, La Jolla, CA.

[4]Tumor Biology Department, H. Lee Moffitt Cancer Center and Research Institute, Tampa, FL.

[5]Department of Anatomic Pathology, H. Lee Moffitt Cancer Center and Research Institute, 12902 Magnolia Drive, Tampa, FL.

[6]Department of Genitourinary Oncology, H. Lee Moffitt Cancer Center and Research Institute, Tampa, FL.

[7]Analytical Genomics and Bioinformatics, Sanford Burnham Prebys Discovery Institute, Orlando, FL.

# Corresponding Author

Kiran Mahajan Ph.D.
Assistant Professor of Surgery
Campus Box 8242
660 South Euclid Avenue
Washington University in St. Louis
St. Louis, MO 63110-1093
Email: kiranm@wustl.edu

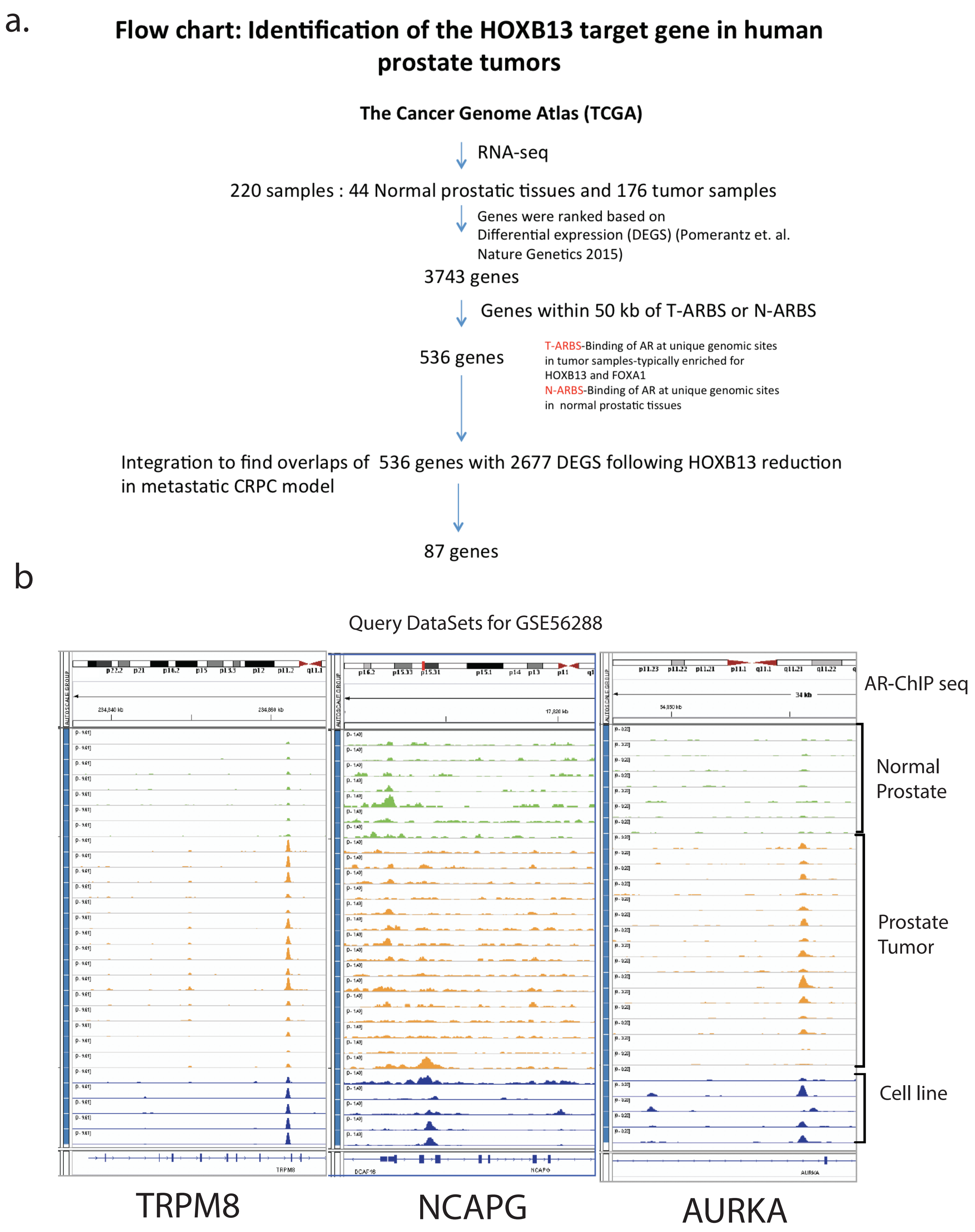
a.
Flow chart: Identification of the HOXB13 target gene in human prostate tumors
The Cancer Genome Atlas (TCGA)
RNA-seq
220 samples : 44 Normal prostatic tissues and 176 tumor samples
Genes were ranked based on Differential expression (DEGS) (Pomerantz et. al. Nature Genetics 2015)
3743 genes
Genes within 50 kb of T-ARBS or N-ARBS
536 genes
T-ARBS-Binding of AR at unique genomic sites in tumor samples-typically enriched for HOXB13 and FOXA1
N-ARBS-Binding of AR at unique genomic sites in normal prostatic tissues
Integration to find overlaps of 536 genes with 2677 DEGS following HOXB13 reduction in metastatic CRPC model
87 genes
b
Query DataSets for GSE56288
AR-ChIP seq
Normal Prostate
Prostate Tumor
Cell line
TRPM8
NCAPG
AURKA

Supplementary Figure 1.

a. Flow chart: Identification of the HOXB13 direct targets in human prostate tumors. Integration of data sets to find overlaps of 536 genes with ~2677 DEGS following HOXB13 reduction in metastatic CRPC model.

b. **IGV analysis for AR and HOXB13 binding sites.** HOXB13 or AR-ChIP sequencing in GSE56288 for binding in the proximity of the putative AR/HOXB13 target genes in normal prostate (1-7 lines, green peaks), human prostate tumors (8-20 lines, orange peaks) and FOXA1 ChIP-seq in tumor (orange peaks- line 21), HOXB13 ChIP-seq tumor (orange peaks- line 22), HOXB13 ChIP-seq in LNCaP (blue peaks- line 23), AR ChIP-seq in normal prostate cell line LHSAR (expressing HOXB13, FOXA1, HOXB13+FOXA1 (#1) HOXB13 +FOXA1 (#2)) (blue peaks lines, 24-27).

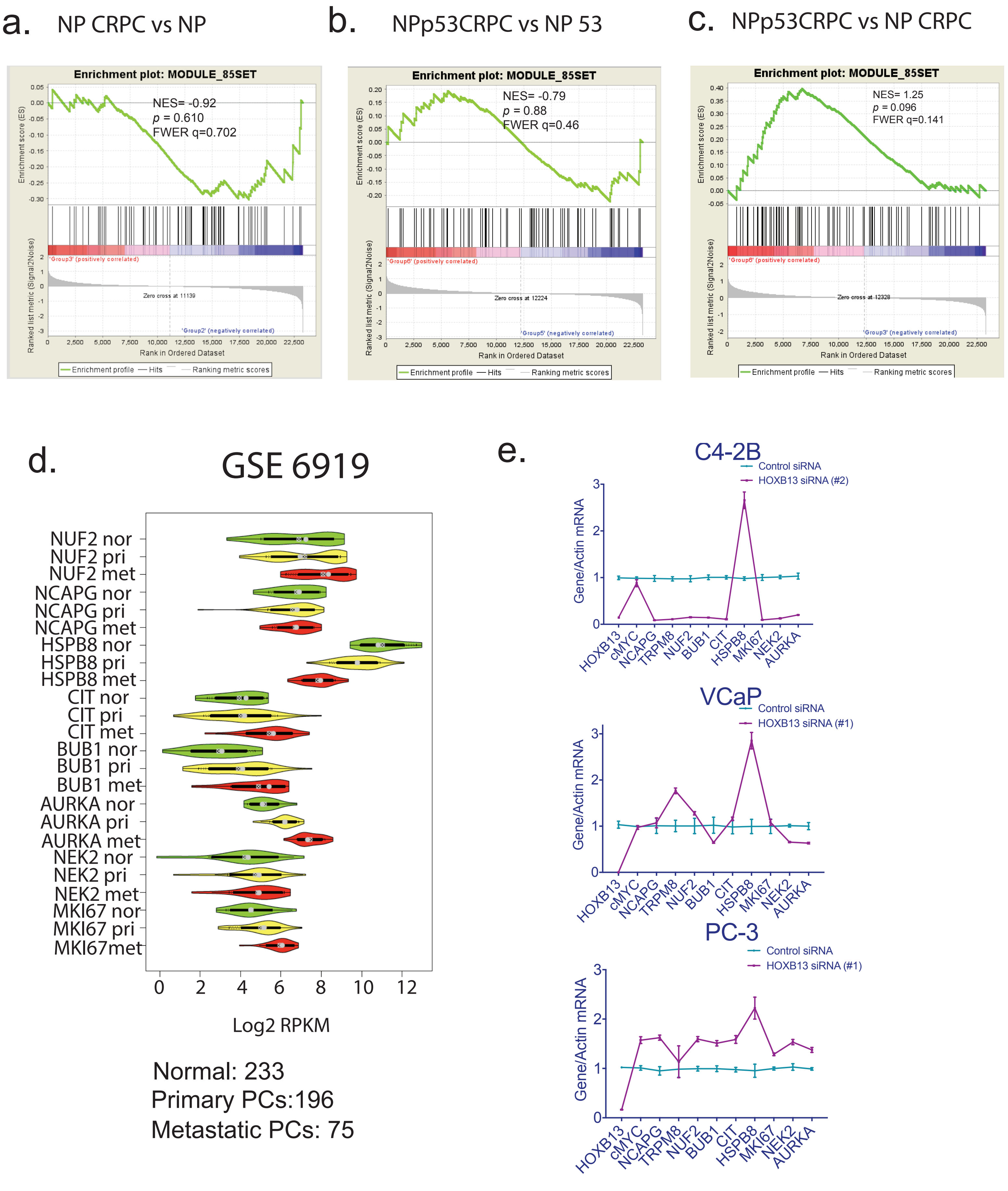

a. NP CRPC vs NP
b. NPp53CRPC vs NP 53
c. NPp53CRPC vs NP CRPC
Enrichment plot: MODULE_85SET
NES= -0.92
p = 0.610
FWER q=0.702
NES= -0.79
p = 0.88
FWER q=0.46
NES= 1.25
p = 0.096
FWER q=0.141
Enrichment score (ES)
Ranked list metric (Signal2Noise)
Rank in Ordered Dataset
Zero cross at 11139
Zero cross at 12224
Zero cross at 12328
Enrichment profile — Hits — Ranking metric scores
d.
GSE 6919
NUF2 nor
NUF2 pri
NUF2 met
NCAPG nor
NCAPG pri
NCAPG met
HSPB8 nor
HSPB8 pri
HSPB8 met
CIT nor
CIT pri
CIT met
BUB1 nor
BUB1 pri
BUB1 met
AURKA nor
AURKA pri
AURKA met
NEK2 nor
NEK2 pri
NEK2 met
MKI67 nor
MKI67 pri
MKI67met
Log2 RPKM
Normal: 233
Primary PCs:196
Metastatic PCs: 75
e.
C4-2B
Control siRNA
HOXB13 siRNA (#2)
VCaP
Control siRNA
HOXB13 siRNA (#1)
PC-3
Control siRNA
HOXB13 siRNA (#1)
Gene/Actin mRNA
HOXB13
cMYC
NCAPG
TRPM8
NUF2
BUB1
CIT
HSPB8
MKI67
NEK2
AURKA

Supplementary Figure 2. HOXB13 transcriptional targets are enriched in high-risk prostate cancers

(a-c) Gene set enrichment analysis comparing HOXB13 target gene expression in intact and castrated (CRPC) *NKX3.1*^{haplo} (N), *NKX3.1*^{haplo} *PTEN*^{cnull} (NP) and *NKX3.1*^{haplo} *PTEN*^{cnull} p53^{cnull} (NPp53) GEMMs of prostate adenocarcinoma. **(d)** Violin plots displaying expression of a subset of HOTPAM9 genes in GSE6919 dataset (Chandran U *et al.,* 2007) comprising normal=76; primary=66 and mPCs=25. *p* values (shown in supplementary table). **(e)** C4-2B, VCaP and PC3 cells were transfected with control or HOXB13 siRNA. Total RNA was isolated, followed by qRT-PCR with HOXB13, c-MYC and HOTPAM9 primers. Actin is used as a normalization control.

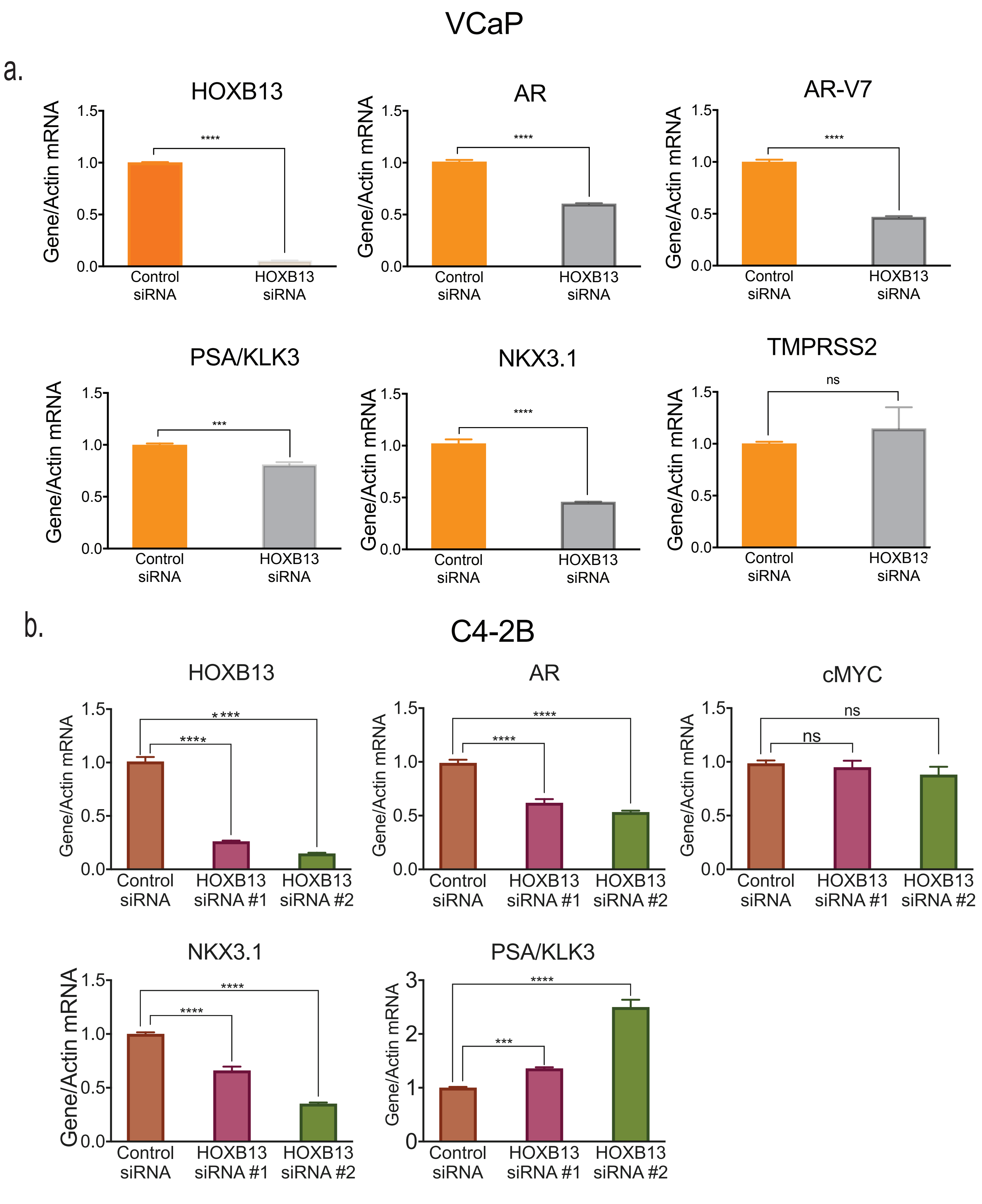
VCaP
a.
HOXB13
AR
AR-V7
PSA/KLK3
NKX3.1
TMPRSS2
Gene/Actin mRNA
Control siRNA
HOXB13 siRNA
****
***
ns
b.
C4-2B
HOXB13
AR
cMYC
NKX3.1
PSA/KLK3
Gene/Actin mRNA
Control siRNA
HOXB13 siRNA #1
HOXB13 siRNA #2

Supplementary Figure 3. HOXB13 regulates expression of AR/AR-V7 in metastatic PCs

(a) VCaP cells were transfected control or HOXB13 siRNA and grown in charcoal-stripped androgen-depleted media. Total RNA was isolated, followed by qRT-PCR with HOXB13, AR, AR-V7 and AR target genes (PSA, TMPRSS2 and NKX3.1) primers. **(b)** C4-2B cells were transfected with control or HOXB13 siRNAs (#1 is individual and #2 is pooled). Total RNA was isolated, followed by qRT-PCR with HOXB13, c-MYC, AR, and AR target genes (PSA and NKX3.1) primers. Data are represented as mean ± SEM and normalized to actin in (a-b). ****$p<0.0001$, ***$p<0.0005$, ns: not significant.

Supplementary Table 1. Association between baseline clinical characteristics with patients disease type for MSKCC data set.

Variable	*Metastatic N=19*	*Primary N=131*	*P-value*
Age at diagnosis (years)			0.15
< 64 Years	12 (63.2%)	103 (78.6%)	
>= 64 Year	7 (36.8%)	28 (21.4%)	
Gleason Score			
5-6	--	41 (31.5%)	
7	--	74 (56.9%)	
8-10	--	15 (11.5%)	
Missing	--	1	
Clinical Tumor Stage			
T1C	--	75 (57.3%)	
T2,T2A,T2B,T2C	--	51 (38.9%)	
T3,T3A,T3B,T3C	--	5 (3.8%)	
Lymph node involvement			<0.001
Abnormal_N1	10 (76.9%)	6 (5.6%)	
Normal_N0	3 (23.1%)	102 (94.4%)	
Missing	6	23	
Margin Status			
Negative	--	100 (76.3%)	
Positive	--	31 (23.7%)	
PSA at Diagnosis			<0.001
< 10	7 (41.2%)	108 (83.1%)	
>= 10	10 (58.8%)	22 (16.9%)	
Missing	2	1	

P-values for categorical variables calculated using the Fisher's Exact method

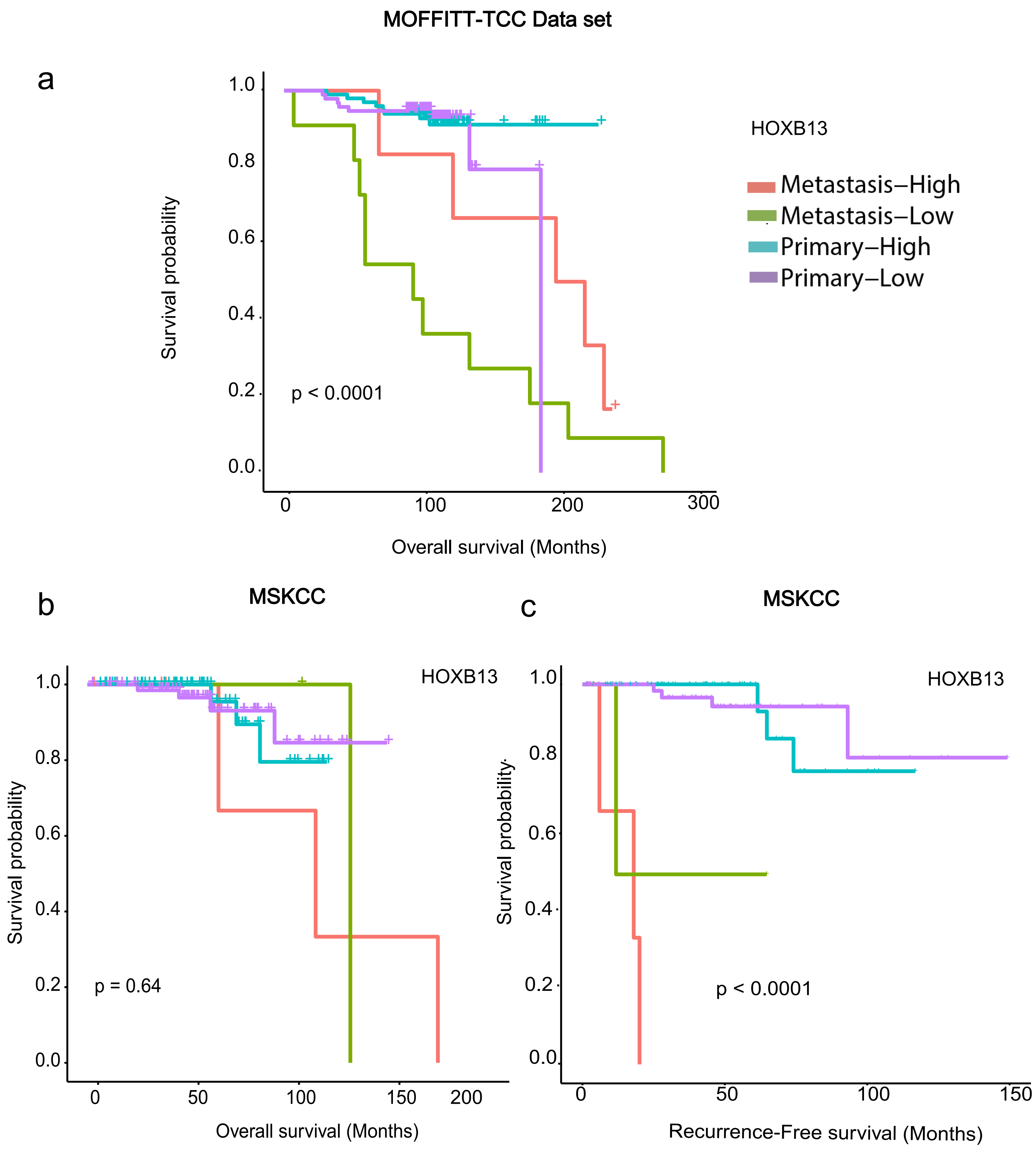
MOFFITT-TCC Data set
a
1.0
0.8
0.6
0.4
0.2
0.0
Survival probability
p < 0.0001
0
100
200
300
Overall survival (Months)
HOXB13
Metastasis–High
Metastasis–Low
Primary–High
Primary–Low
b
MSKCC
HOXB13
1.0
0.8
0.6
0.4
0.2
0.0
Survival probability
p = 0.64
0
50
100
150
200
Overall survival (Months)
c
MSKCC
HOXB13
1.0
0.8
0.6
0.4
0.2
0.0
Survival probability
p < 0.0001
0
50
100
150
Recurrence-Free survival (Months)

Supplementary Figure 4. Kaplan-Meier overall survival analysis evaluating *HOXB13* gene expression in human prostate cancers

(a) Moffitt TCC data set–overall log rank survival ($p<0.0001$) and **(b)** MSKCC data set-overall log rank survival ($p=0.64$) and **(c)** MSKCC data set-Recurrence Free Survival ($p<0.0001$).

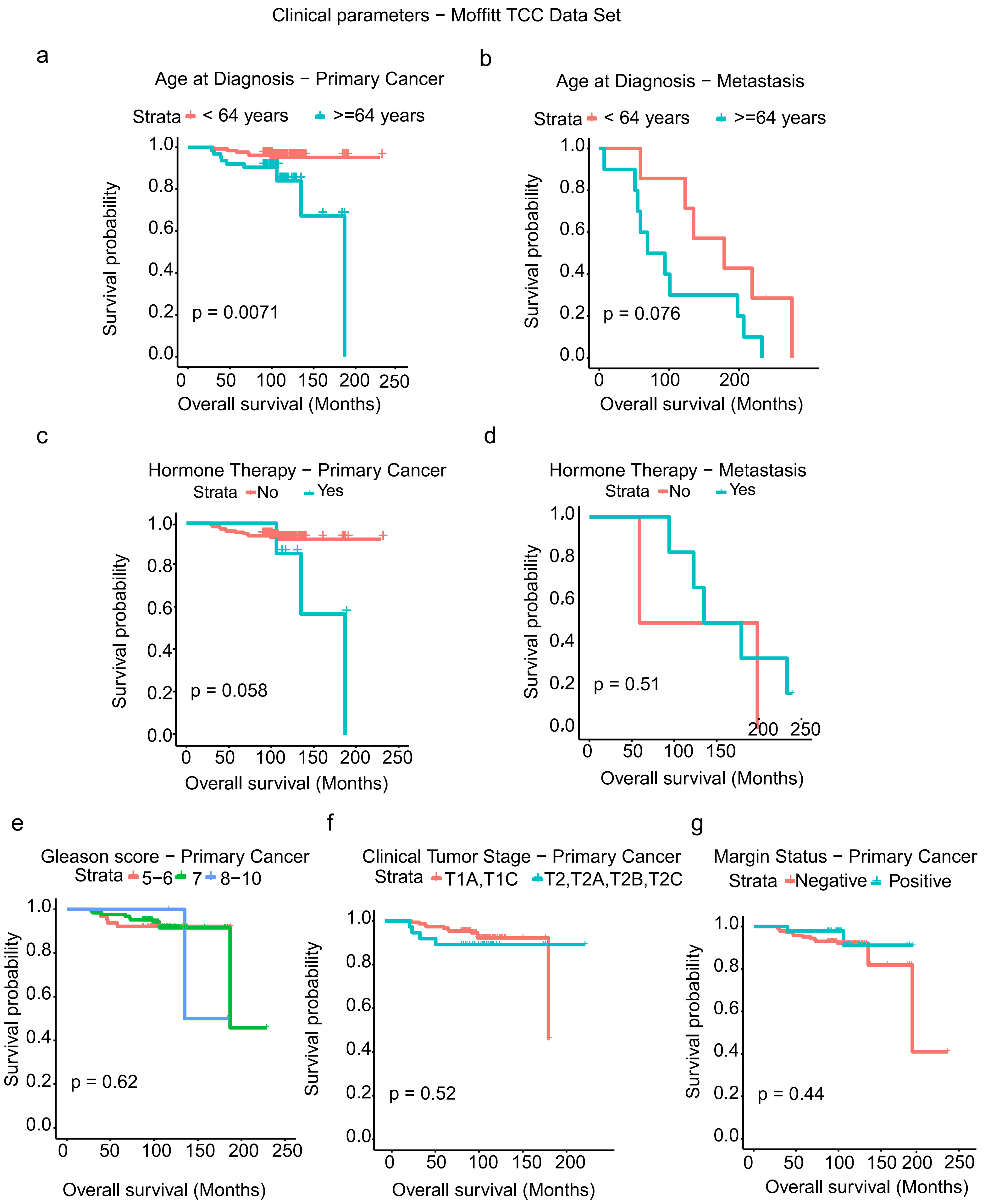

Clinical parameters − Moffitt TCC Data Set
a
Age at Diagnosis − Primary Cancer
Strata < 64 years >=64 years
Survival probability
1.0
0.8
0.6
0.4
0.2
0.0
p = 0.0071
0 50 100 150 200 250
Overall survival (Months)
b
Age at Diagnosis − Metastasis
Strata < 64 years >=64 years
Survival probability
p = 0.076
0 100 200
Overall survival (Months)
c
Hormone Therapy − Primary Cancer
Strata No Yes
Survival probability
p = 0.058
0 50 100 150 200 250
Overall survival (Months)
d
Hormone Therapy − Metastasis
Strata No Yes
Survival probability
p = 0.51
0 50 100 150 200 250
Overall survival (Months)
e
Gleason score − Primary Cancer
Strata 5−6 7 8−10
Survival probability
p = 0.62
0 50 100 150 200 250
Overall survival (Months)
f
Clinical Tumor Stage − Primary Cancer
Strata T1A,T1C T2,T2A,T2B,T2C
Survival probability
p = 0.52
0 50 100 150 200
Overall survival (Months)
g
Margin Status − Primary Cancer
Strata Negative Positive
Survival probability
p = 0.44
0 50 100 150 200 250
Overall survival (Months)

Supplementary Figure 5. Survival analysis of Moffitt TCC data set-Clinical parameters.

(**a-g**) Kaplan-Meier overall survival curves based on categorized gene expression levels for HOTPAM9 genes in the Moffitt TCC data set. The following parameters were considered: age at diagnosis for primary and metastatic prostate cancer, hormone therapy for primary and metastatic prostate cancer, Gleason score, clinical tumor stage and margin status for primary disease. *p* values were generated by the Log-rank test and univariate Cox proportional hazards regression models.

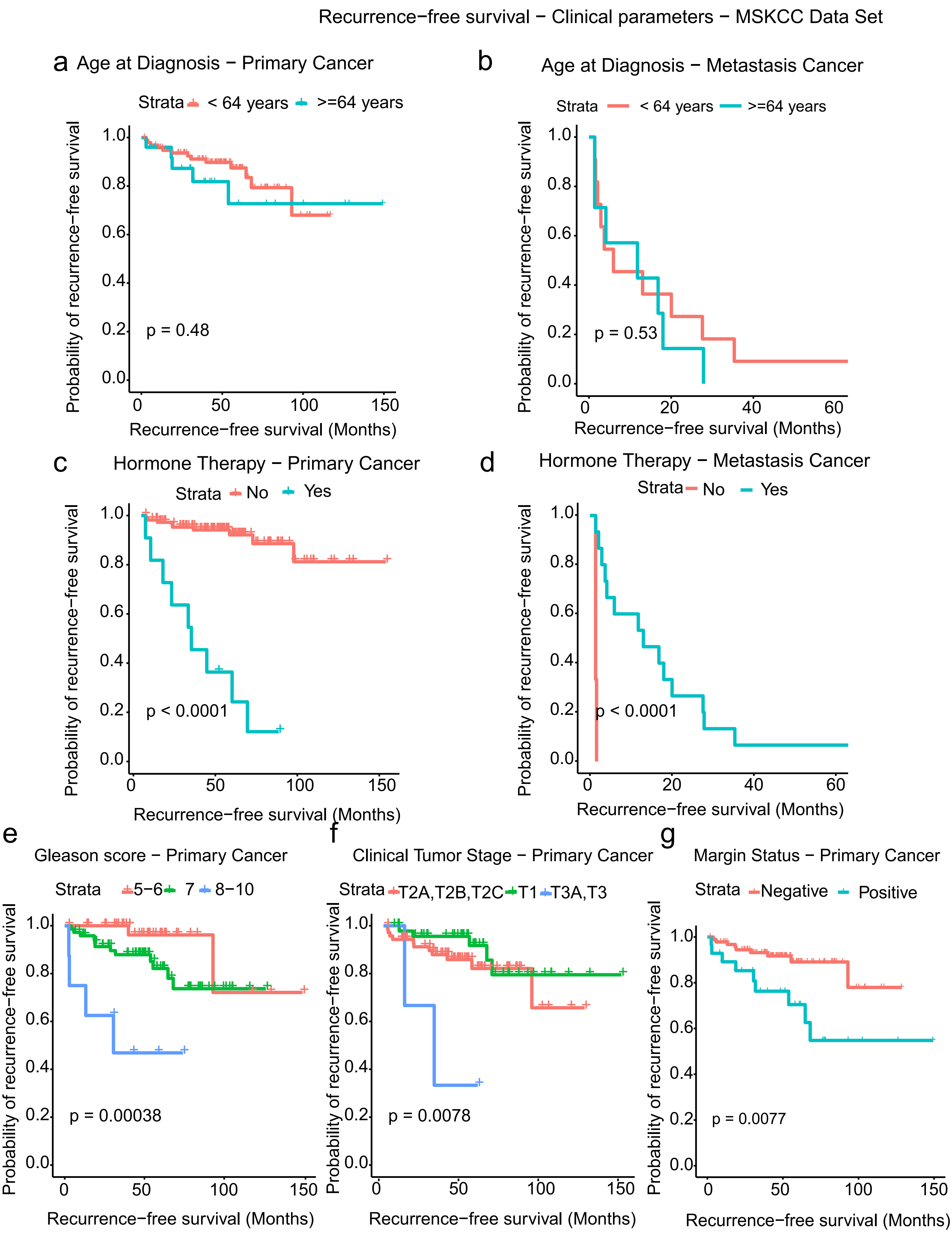

Recurrence−free survival − Clinical parameters − MSKCC Data Set
a Age at Diagnosis − Primary Cancer
Strata < 64 years >=64 years
p = 0.48
b Age at Diagnosis − Metastasis Cancer
Strata < 64 years >=64 years
p = 0.53
c Hormone Therapy − Primary Cancer
Strata No Yes
p < 0.0001
d Hormone Therapy − Metastasis Cancer
Strata No Yes
p < 0.0001
e Gleason score − Primary Cancer
Strata 5−6 7 8−10
p = 0.00038
f Clinical Tumor Stage − Primary Cancer
Strata T2A,T2B,T2C T1 T3A,T3
p = 0.0078
g Margin Status − Primary Cancer
Strata Negative Positive
p = 0.0077
Probability of recurrence−free survival
Recurrence−free survival (Months)

Supplementary Figure 6. Kaplan-Meier Recurrence Free Survival (RFS) analysis of MSKCC data set - clinical parameters.

(**a-g**) Kaplan-Meier RFS survival curves based on categorized gene expression levels for HOTPAM9 genes in the MSKCC data set. The following parameters were considered: age at diagnosis for primary and metastatic prostate cancer, hormone therapy for primary and metastatic prostate cancer, Gleason score, clinical tumor stage and margin status for primary disease. *p* values were generated by the log-rank test.

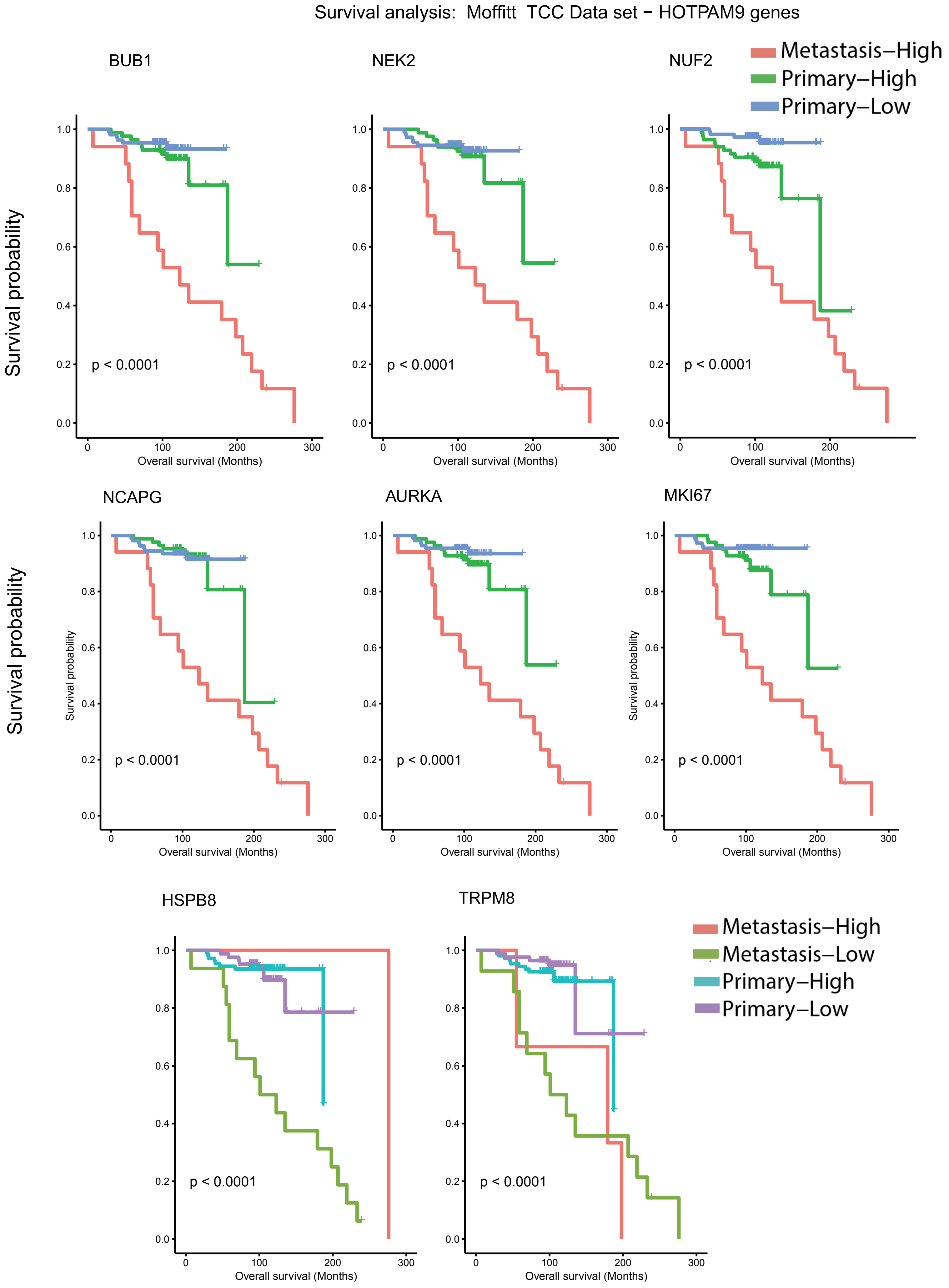
Survival analysis: Moffitt TCC Data set − HOTPAM9 genes
BUB1
NEK2
NUF2
Metastasis−High
Primary−High
Primary−Low
Survival probability
p < 0.0001
Overall survival (Months)
NCAPG
AURKA
MKI67
HSPB8
TRPM8
Metastasis−High
Metastasis−Low
Primary−High
Primary−Low

Supplementary Figure 7. Kaplan-Meier overall survival analysis of HOTPAM9 gene set in Moffitt TCC data Set. Kaplan-Meier overall survival curves based on categorized gene expression levels of HOTPAM9 genes in Moffitt TCC data set. *p* values were generated by the log-rank test.

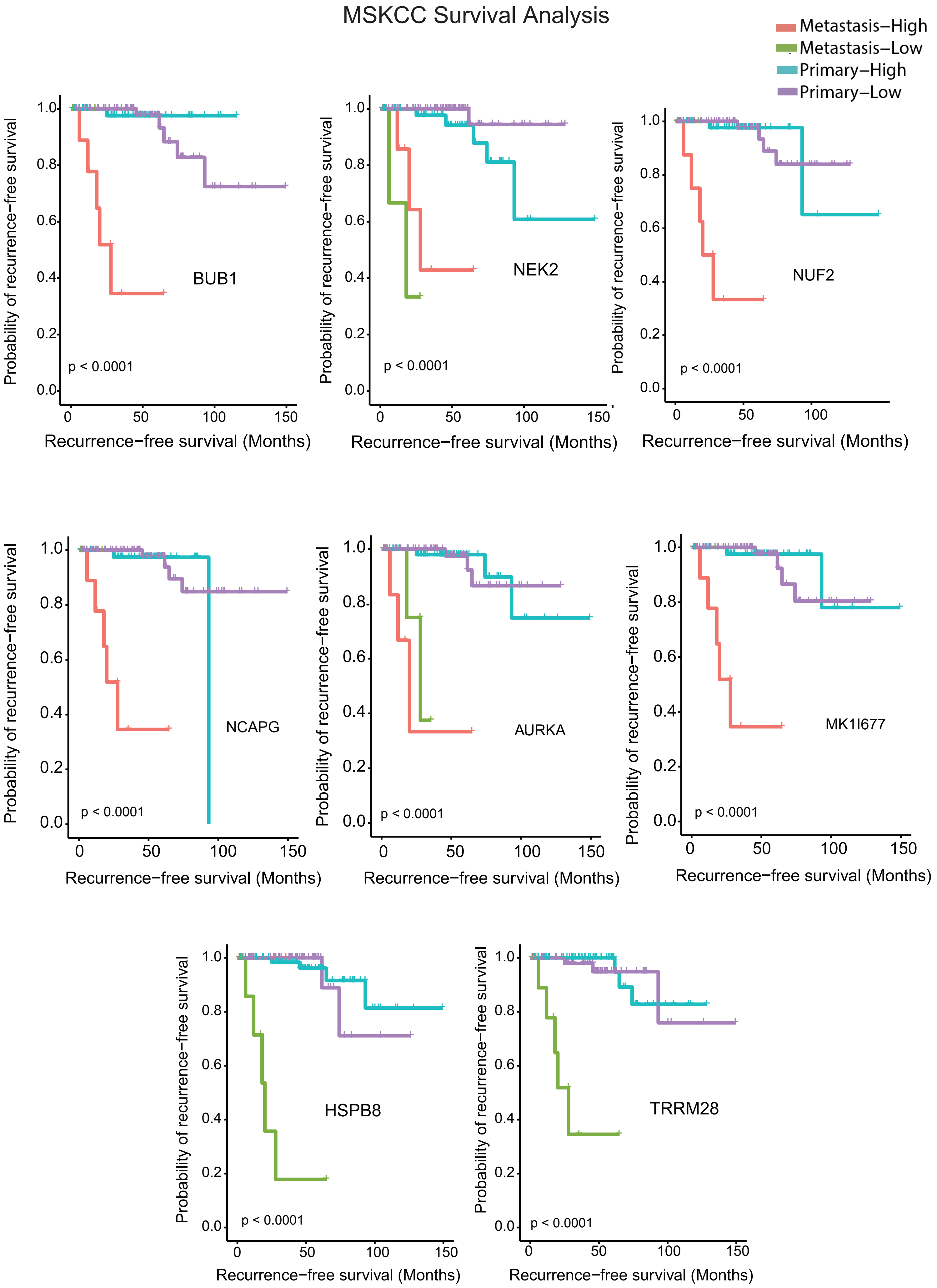
MSKCC Survival Analysis
Metastasis−High
Metastasis−Low
Primary−High
Primary−Low
BUB1
NEK2
NUF2
NCAPG
AURKA
MK1I677
HSPB8
TRRM28
p < 0.0001
Probability of recurrence−free survival
Recurrence−free survival (Months)
0.0
0.2
0.4
0.6
0.8
1.0
0
50
100
150

Supplementary Figure 8. Survival analysis evaluating HOTPAM9 gene expression in MSKCC data set. Kaplan-Meier Recurrence Free survival curves based on categorized gene expression levels of HOTPAM9 genes in MSKCC data set. *p* values were generated by the log-rank test.

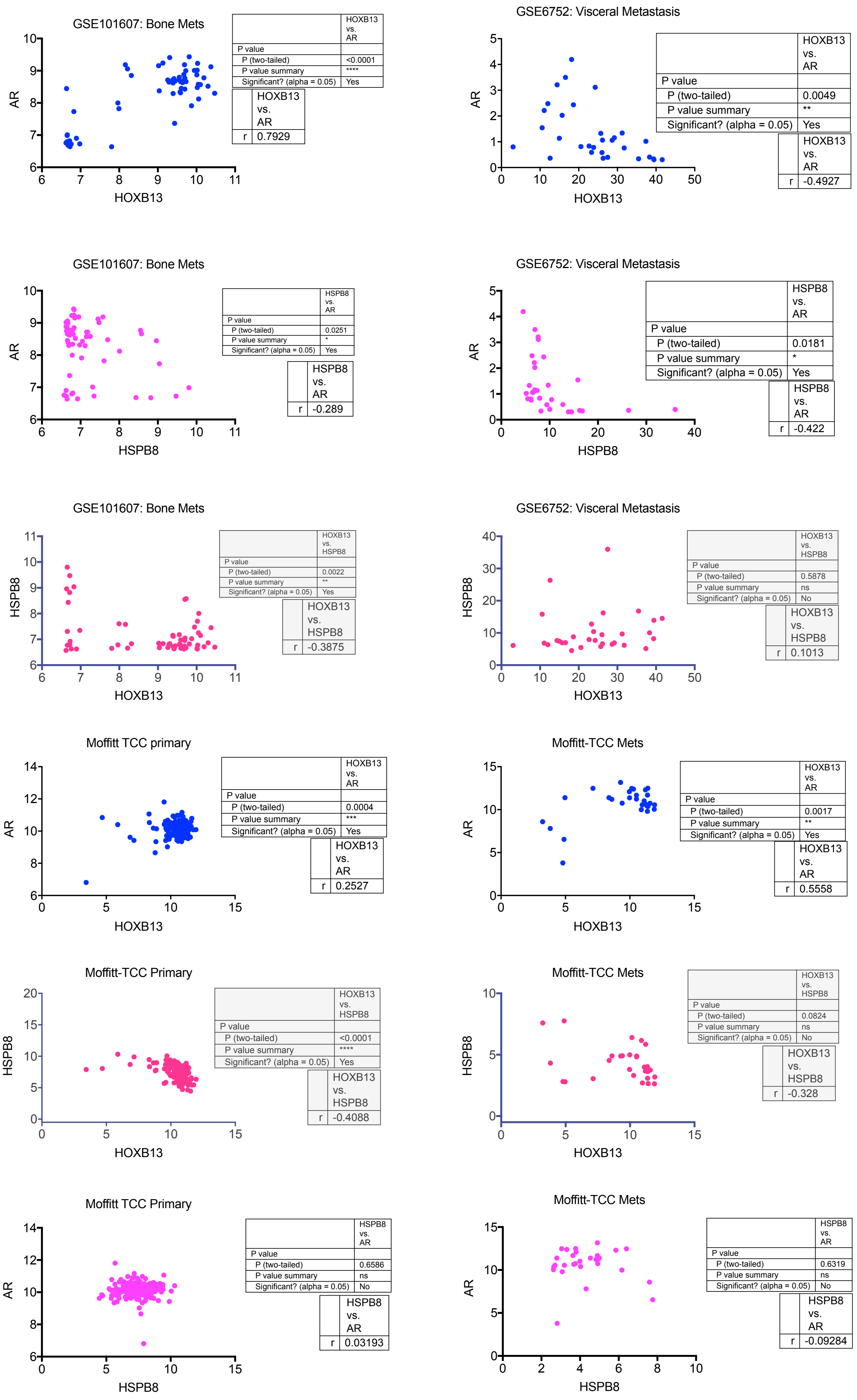
GSE101607: Bone Mets
AR
HOXB13
HOXB13 vs. AR
P value
P (two-tailed) <0.0001
P value summary ****
Significant? (alpha = 0.05) Yes
r 0.7929
GSE6752: Visceral Metastasis
AR
HOXB13
HOXB13 vs. AR
P value
P (two-tailed) 0.0049
P value summary **
Significant? (alpha = 0.05) Yes
r -0.4927
GSE101607: Bone Mets
AR
HSPB8
HSPB8 vs. AR
P value
P (two-tailed) 0.0251
P value summary *
Significant? (alpha = 0.05) Yes
r -0.289
GSE6752: Visceral Metastasis
AR
HSPB8
HSPB8 vs. AR
P value
P (two-tailed) 0.0181
P value summary *
Significant? (alpha = 0.05) Yes
r -0.422
GSE101607: Bone Mets
HSPB8
HOXB13
HOXB13 vs. HSPB8
P value
P (two-tailed) 0.0022
P value summary **
Significant? (alpha = 0.05) Yes
r -0.3875
GSE6752: Visceral Metastasis
HSPB8
HOXB13
HOXB13 vs. HSPB8
P value
P (two-tailed) 0.5878
P value summary ns
Significant? (alpha = 0.05) No
r 0.1013
Moffitt TCC primary
AR
HOXB13
HOXB13 vs. AR
P value
P (two-tailed) 0.0004
P value summary ***
Significant? (alpha = 0.05) Yes
r 0.2527
Moffitt-TCC Mets
AR
HOXB13
HOXB13 vs. AR
P value
P (two-tailed) 0.0017
P value summary **
Significant? (alpha = 0.05) Yes
r 0.5558
Moffitt-TCC Primary
HSPB8
HOXB13
HOXB13 vs. HSPB8
P value
P (two-tailed) <0.0001
P value summary ****
Significant? (alpha = 0.05) Yes
r -0.4088
Moffitt-TCC Mets
HSPB8
HOXB13
HOXB13 vs. HSPB8
P value
P (two-tailed) 0.0824
P value summary ns
Significant? (alpha = 0.05) No
r -0.328
Moffitt TCC Primary
AR
HSPB8
HSPB8 vs. AR
P value
P (two-tailed) 0.6586
P value summary ns
Significant? (alpha = 0.05) No
r 0.03193
Moffitt-TCC Mets
AR
HSPB8
HSPB8 vs. AR
P value
P (two-tailed) 0.6319
P value summary ns
Significant? (alpha = 0.05) No
r -0.09284

Supplementary Figure 9. Low levels of HSPB8 mRNA expression negatively correlate with increased HOXB13 expression in PCs

Pearson correlation analysis was performed on multiple metastatic PC data sets (GSE101607, GSE6752, GSE67980 and Moffit TCC data sets) to examine the correlation between AR, HOXB13 and HSPB8 expression in PCs metastasized to the bone, lymph node, lung, liver and other organs. Pearson regression co-efficient (r) values and p values are shown in the tables.

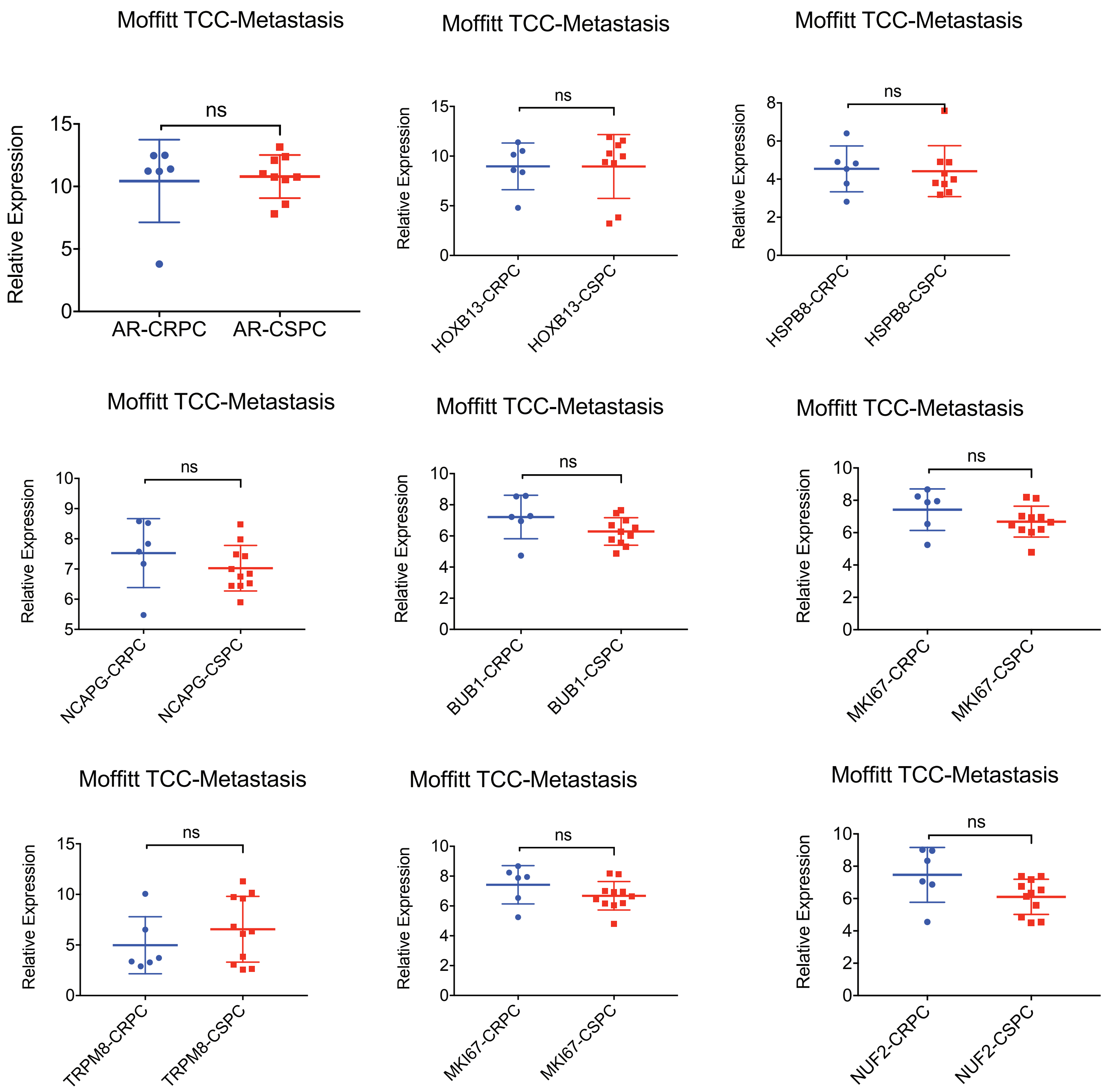

Moffitt TCC-Metastasis
Relative Expression
ns
AR-CRPC
AR-CSPC
HOXB13-CRPC
HOXB13-CSPC
HSPB8-CRPC
HSPB8-CSPC
NCAPG-CRPC
NCAPG-CSPC
BUB1-CRPC
BUB1-CSPC
MKI67-CRPC
MKI67-CSPC
TRPM8-CRPC
TRPM8-CSPC
NUF2-CRPC
NUF2-CSPC

Supplementary Figure 10. Expression of HOTPAM9 genes in metastatic PC samples pre- and post-anti-androgen therapies. Relative expression of HOTPAM9 genes pre- and post-castration treatment in the Moffitt TCC data set. ns: not significant. The metastatic samples were obtained from multiple sites (lung, liver, bone, lymph nodes and soft tissue), while primary samples were obtained from anterior/posterior/apex/mid/base of prostate.

Uncropped images of Western blots presented in Figure 3a, 8a, 8b

Figure 3a

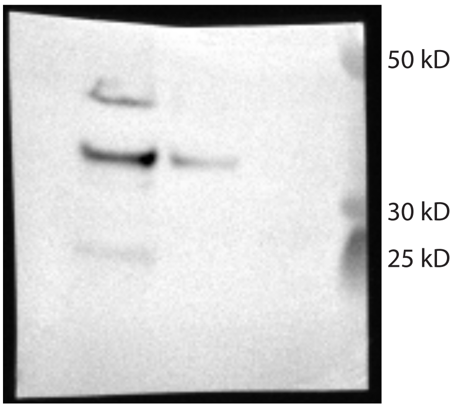

HOXB13

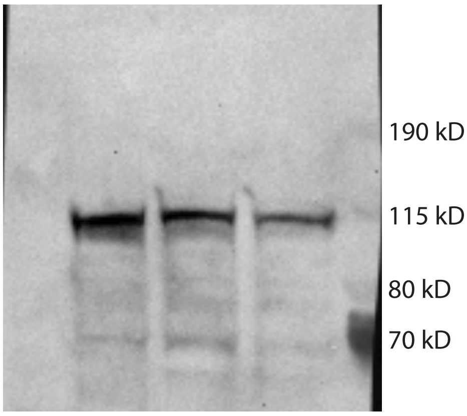

AR

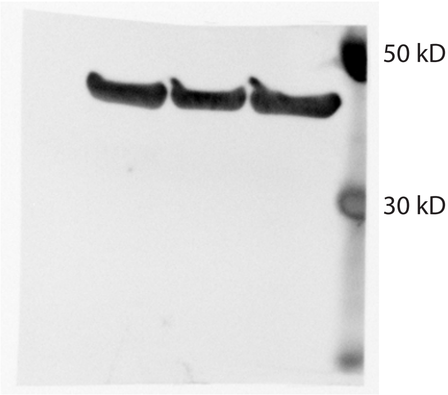

Actin

Figure 8a

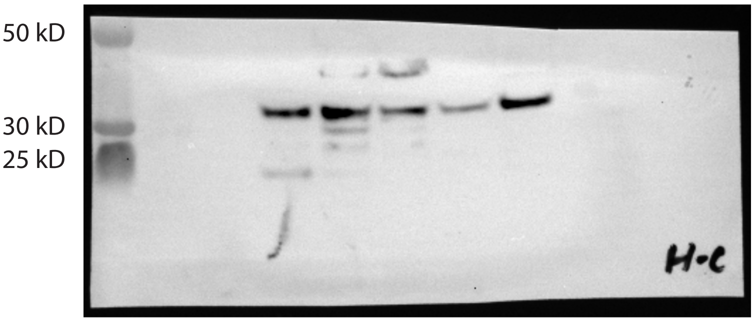

HOXB13

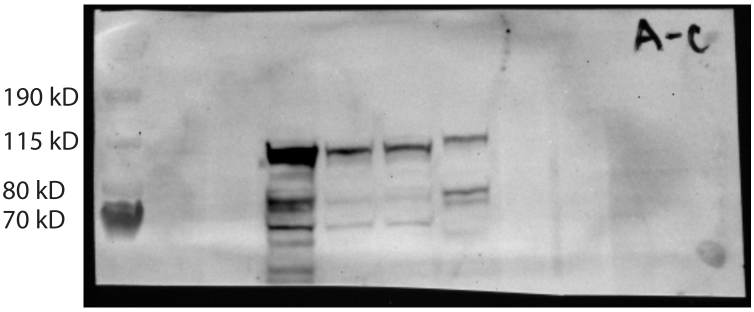

AR

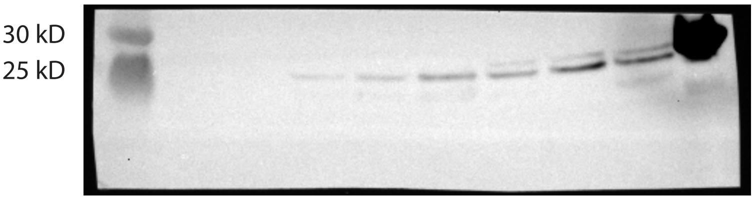

HSPB8

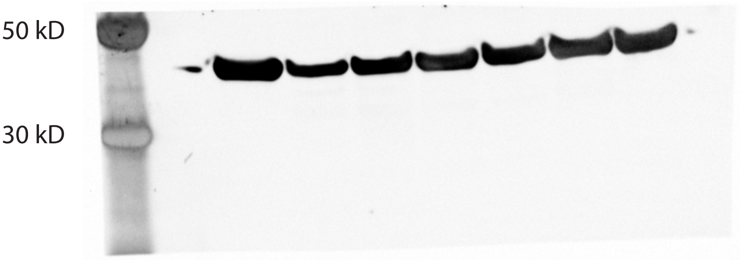

Actin

Figure 8b

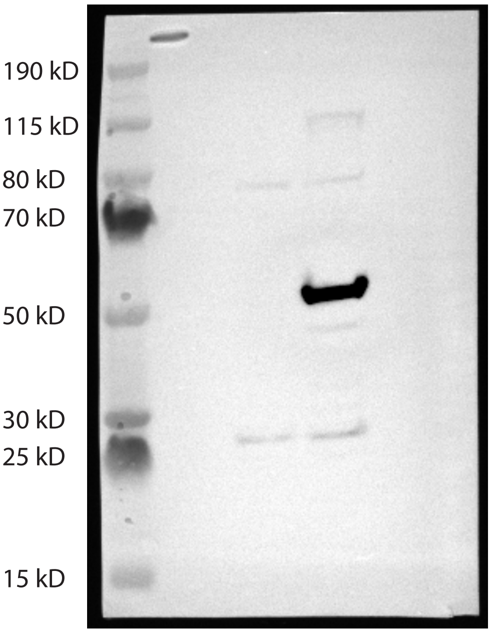

HSPB8

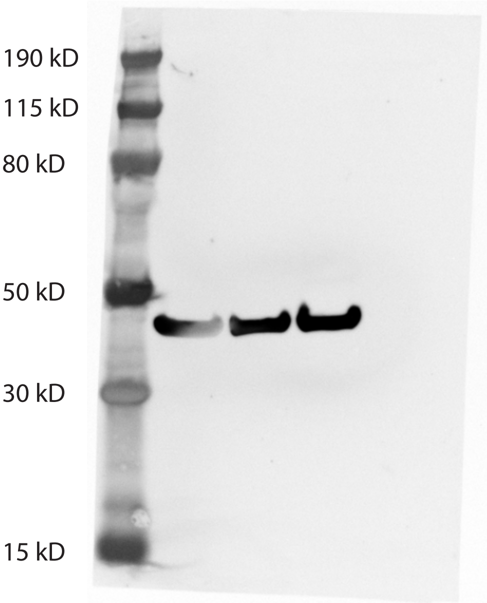

Actin
